# CVM-1118 (foslinanib), a 2-phenyl-4-quinolone derivative, promotes apoptosis and inhibits vasculogenic mimicry via targeting TRAP1

**DOI:** 10.3389/pore.2023.1611038

**Published:** 2023-06-07

**Authors:** Lifen Shen, Yen-Ling Chen, Chu-Chun Huang, Yu-Chiau Shyu, Richard E. B. Seftor, Elisabeth A. Seftor, Mary J. C. Hendrix, Du-Shieng Chien, Yi-Wen Chu

**Affiliations:** ^1^ TaiRx, Inc., Taipei, Taiwan; ^2^ Community Medicine Research Center, Chang Gung Memorial Hospital Keelung Branch, Keelung, Taiwan; ^3^ Department of Nursing, Chang Gung University of Science and Technology, Taoyuan, Taiwan; ^4^ Department of Biology, Shepherd University, Shepherdstown, WV, United States

**Keywords:** TRAP1, vasculogenic mimicry, CVM-1118, foslinanib, Hsp75, *STK11*, *NF2*

## Abstract

CVM-1118 (foslinanib) is a phosphoric ester compound selected from 2-phenyl-4-quinolone derivatives. The NCI 60 cancer panel screening showed CVM-1125, the major active metabolite of CVM-1118, to exhibit growth inhibitory and cytotoxic effects at nanomolar range. CVM-1118 possesses multiple bioactivities, including inducing cellular apoptosis, cell cycle arrest at G_2_/M, as well as inhibiting vasculogenic mimicry (VM) formation. The TNF receptor associated protein 1 (TRAP1) was identified as the binding target of CVM-1125 using nematic protein organization technique (NPOT) interactome analysis. Further studies demonstrated CVM-1125 reduced the protein level of TRAP1 and impeded its downstream signaling by reduction of cellular succinate levels and destabilization of HIF-1α. The pharmacogenomic biomarkers associated with CVM-1118 were also examined by Whole Genome CRISPR Knock-Out Screening. Two hits (*STK11* and *NF2*) were confirmed with higher sensitivity to the drug in cell knock-down experiments. Biological assays indicate that the mechanism of action of CVM-1118 is via targeting TRAP1 to induce mitochondrial apoptosis, suppress tumor cell growth, and inhibit vasculogenic mimicry formation. Most importantly, the loss-of-function mutations of *STK11* and *NF2* are potential biomarkers of CVM-1118 which can be applied in the selection of cancer patients for CVM-1118 treatment. CVM-1118 is currently in its Phase 2a clinical development.

## Introduction

CVM-1118 (generic name: foslinanib) is a phosphoric ester selected from a series of synthetic 2-phenyl-4-quinolone analogues which contain phenol-quinoline chemophore as the core structure required to exhibit anti-neoplastic and anti-mutagenic properties [1–9]. In humans and animal species (e.g., mouse, rat, dog, monkey), CVM-1118 is rapidly and completely metabolized via dephosphorylation to form CVM-1125 following either intravenous or oral administration. Their anti-cancer activities have been tested using a panel of 60 human cancer cell lines (NCI 60 screen). The results showed that 87% cell lines tested had an average GI_50_ value < 100 nM. CVM-1125 inhibits cell proliferation, while induces apoptosis in human melanoma cells. In addition, CVM-1118 exhibits potent inhibition of vasculogenic mimicry (VM) [10]. Currently, this novel chemical entity (NCE) is being developed as an oral anti-cancer drug.

VM describes the plasticity of aggressive cancer cells forming *de novo* vascular perfusion networks, which is distinct from traditional endothelial tumor angiogenesis [11, 12]. The VM phenotype is known to correlate with malignant characteristics, invasion, tumor progression, metastasis, and worse survival outcome in cancer patients [13]. Angiogenesis inhibitors, such as bevacizumab, a monoclonal antibody against vascular endothelial growth factor A (VEGF-A), are ineffective in reducing VM, which may contribute to drug resistance in cancer cells [14, 15]. Until now, there are no inhibitors targeting VM on the drug market; therefore, development of anti-VM compounds is considered a novel approach for next-generation cancer treatment.

Tumor necrosis factor receptor associated protein 1 (TRAP1), also known as Hsp75, is a molecular chaperon localized in the mitochondria and is a member of the heat shock protein family [16, 17]. It has been reported that TRAP1 has multiple cellular functions, e.g., antagonizing mitochondrial apoptosis by regulating mitochondrial permeability transition pores, antagonizing mitochondrial oxidative stress, and promoting drug resistance [18–21]. Recently, the role of TRAP1 has also been linked to reprogramming metabolism by inhibiting oxidative phosphorylation and switching to aerobic glycolysis [22]. TRAP1 is highly expressed in many types of human tumors, including breast cancer, hepatocellular carcinoma (HCC), and glioblastoma [23–25]. Its expression is correlated with tumor stage, and high expression of TRAP1 correlates with poor survival in colon cancer and NSCLC [26, 27].

In this report, we present experimental evidence demonstrating the mechanism of action of CVM-1118 as a novel anti-cancer drug. The results show that CVM-1118 targets multiple cellular processes, including induction of cell apoptosis and cell cycle arrest. In addition, CVM-1118 inhibits the formation of VM *in vitro* and *in vivo*. Moreover, through nematic protein organization technique (NPOT) and further analysis by surface plasmon resonance (SPR), TRAP1 was identified as one of the cellular binding targets of CVM-1118. Translating the discovery of the binding between CVM-1118 and TRAP1 into a functional cellular activity, it was found that one of the impacts of CVM-1118 on TRAP1 was affecting its protein level by lysosomal degradation. These results demonstrate CVM-1118 as a potential novel anti-cancer drug, which functions to inhibit tumor growth and VM formation and induce cancer cell apoptosis via targeting TRAP1. The potential pharmacogenomic biomarkers for CVM-1118 were further identified which may be applied in cancer patient selection for future clinical investigation of CVM-1118.

## Materials and methods

### Cell culture

For cytotoxicity and cell cycle analysis, the following cell lines were used, and their culture conditions were as described: for A549, DU-145, HT-29, MDA-MB-231, Mia PaCa-2, and U118MG, cells were cultured in DMEM containing 10% FCS; for MDA- MB-435 and NCI-ADR-RES, cells were cultured in RPMI-1640 containing 10% FCS, and for SK-N-MC, cells were cultured in MEM containing 10% FCS. For the animal pharmacology study, HCT-116, a colon cancer cell line was tested. Cells were cultured in McCoy’s 5a medium supplemented with 10% heat inactivated fetal bovine serum. For the cell lines used in VM analysis and target identification, C8161 and COLO205 cells were maintained in RPMI-1640 containing 10% FBS and 0.1% gentamycin sulfate. The SKOV3 line was cultured in RPMI-1640 supplemented with 15% FBS and 0.1% gentamycin sulfate. For the SK-MEL28 and MCF-7, cells were maintained in MEM/Earles Salts supplemented with 10% FBS and 0.1% gentamycin sulfate.

### Cytotoxicity assays

This assay was conducted in ProQinase GmbH (Freiburg, Germany). Nine cancer cell lines (A549, DU-145, HT-29, MDA-MB-231, MDA-MB-435, Mia PaCa-2, NCI-ADR-RES, SK-N-MC, and U118MG) were seeded at 5,000 cells/well in 96-well cell culture plates in 150 µL of complete medium for 24 h prior to treatment. The test compounds CVM-1118 and CVM-1125 were synthesized by China Medical University, Chengdu Chempartner Co., Ltd., and Formosa Laboratories, Inc. To prepare the assay, the compounds were dissolved in 100% DMSO at a concentration of 10 μM. Cells were then treated with vehicle, CVM-1118 or CVM-1125 at various concentrations for 72 h. Cell viability was determined by the fluorescent quantification of Alamar Blue (Invitrogen, Thermo Fisher Scientific). By the end of incubation, Alamar Blue reagent was added to the well for 4 h and the fluorescence was measured at 590 nm. The amount of fluorescence produced is proportional to the number of metabolically active cells. IC_50_ determination was performed using a variable slope sigmoidal response fitting model with 0% cell growth as a bottom constraint and 100% cell growth as a top constraint (GraphPad Prism, United States).

### Flow cytometry analysis

This assay was conducted by ProQinase GmbH (Freiburg, Germany). HT-29 colorectal adenocarcinoma cells were seeded at 150,000 cells/well in 6-well cell culture plates in 5 mL complete medium and incubated at 37°C overnight. On the day of the assay, CVM-1118 at various concentrations or 0.1% DMSO were added to the cells. After 48 h incubation, cells were harvested including the supernatant, fixed in 80% methanol, and stored at −20°C overnight. The next day, cells were rehydrated in PBS for 30 min and treated with RNAse A (100 μg/mL) and propidium iodide (PI) (25 μg/mL). Cell cycle distribution was analyzed by flow cytometry (FACSCalibur, BD Biosciences), assessing approximately 10,000 events. Detection of DNA intercalated with PI was done by fluorescence detector FL3, in which amplification was set in such a manner that the first amplitude peak of the FL3 signal (FL3-A) in untreated cells (corresponding to the G_1_ cell population) was found at approximately 200 units. For analysis, events were plotted as histogram. The peak at ∼200 units was defined as cells in G_1_, events below the G_1_-peak were considered apoptotic cells (sub-G_1_), and events at ∼400 units as G_2_/M cells. All signals in between 200 and 400 units were defined as cells in S-Phase, and those beyond 400 units as endoreduplicated cells (endoR).

### *In vitro* VM assay

Aggressive human melanoma (C8161) cells were seeded at 1.0 × 10^5^ onto 3D polymerized LDEV-free Matrigel (75 μL; 14 mg/ml; Corning Cat. No. 356232) for VM analysis in 12-well dishes containing no drug or various concentrations of CVM-1118. Matrigel-coated dishes were photographed for tubular network formation after 24 h.

### Orthotopic HCT-116 mouse xenograft study and *in vivo* vasculogenic mimicry

This animal study was contracted to CrownBio International R&D Center (Beijing, China) and was conducted under the approval of the Animal Ethics Committee at CrownBio in compliance with institutional and national guidelines. Seven-to eight-week-old female BALB/c nude mice were obtained from HFK Bio-Technology Co., Ltd. (Beijing, China). HCT-116 cells (2 × 10^6^) were suspended in 20 μL PBS and then injected into the cecal wall. Treatment began on day 14 after cell inoculation when tumor reached ∼100 mm^3^ measured in a satellite group of three mice. Treatment groups include vehicle control [PEG400:pH 10 carbonate buffer (40:60)], CVM-1118 at 20, 50, and 100 mg/kg given orally daily for 28 days, and CVM-1118 at 20 mg/kg given as intravenous injection once every 2 days for 7 times. Tumor volumes were measured at the end of in-life study on day 43. At the end of *in vivo* study, tumor tissues from selected groups were excised, collected, and subjected to standard immunohistochemical procedures to visualize the effect of CVM-1118 on VM network formation. Briefly, rabbit anti-human CD31 antibody (Abcam) was used as the primary Ab for the staining and the number of channels with CD31+/red blood cell (RBC)+, or CD31−/RBC+ were counted based on five light fields (×200) on the whole section of each sample in each group under microscope. Analysis of difference in tumor volume for drug-treated group against vehicle control group was conducted using one-way ANOVA followed by least significant difference (LSD) test (equal variance tested and confirmed by Brown-Forsythe test). Statistical analysis for VM formation was evaluated using Mann-Whitney test. All data were analyzed using GraphPad Prism 9.5.

### Nematic protein organization technique (NPOT) and *in silico* target interaction studies

This assay was contracted to Inoviem Scientific (Strasbourg, France). The NPOT proprietary technology is based on the Kirkwood-Buff molecular crowding and aggregation theory [28, 29]. COLO205 and MCF7 cell lines as well as human primary colorectal cancer tissues and human melanoma cancer tissues were used for the study. The colorectal cancer tissues were obtained from the tumor bank of the Mannheim Medical Faculty, University of Heidelberg, Germany. The biobank was approved by the Ethical Committee of this institution. The melanoma tissues were obtained from Strasbourg University Hospital, France. The sample collection was approved by the Commission Nationale Informatique et Liberté and the Institutional Board of Strasbourg University Hospital. All the enrolled subjects provided written informed consent and were collected through protocols conforming to ethical requirements. Cell or tissue lysates were prepared under low temperature (4°C) in the absence of any detergents, reducing agents and protease or phosphatase inhibitors. All dilutions and washes were performed in standard 4- (2-hydroxyethyl)-1-piperazine ethanesulfonic acid (HEPES)-buffered saline solution (HBSS) unless otherwise indicated. NPOT was performed as described elsewhere [30–32]. Briefly, under sterile conditions at 4°C, 1 µM of CVM-1125 was mixed separately with 1 µg of COLO205 and MCF7 cell lysates, or human colorectal and melanoma tumor homogenates which contain both cytoplasmic and membrane proteins, and then subjected to NPOT isolation. Experiments were performed three times independently. After NPOT isolation, the heteroassemblies were allowed to form overnight, and captured in 96-well plates in the form of a droplet on the surface of mineral oil, and then isolated by microdissection under a microscope. Isolated heteroassemblies were washed in acetone, solubilized in HBSS, then filtered through a 10% sodium dodecyl sulfate polyacrylamide gel electrophoresis (SDS-PAGE) gel composed of 5 mm stacking and 5 mm running gels. Heteroassemblies after SDS-PAGE isolation were solubilized in 10 µL of 2D buffer (7 M urea, 2 M thiourea, 4% CHAPS, 20 mM DTT, 1 mM PMSF). Proteins were precipitated in acetate buffer by centrifugation for 20 min at 7500xg. Thereafter, pellets were digested for 1 h with trypsin Gold (Promega) at 37°C. The samples were resuspended at 1 μg/μL in 50 mM acetic acid, and then diluted in 40 mM NH_4_HCO_3_ to 20 μg/mL. The samples were then dried in SpeedVac^®^ at room temperature and subsequently purified and concentrated using ZipTip^®^ pipette tips (Millipore Corporation) before being subjected to LC-MS/MS using ESI-QUAD-TOF machine. Proteins were identified using Mascot software.

To confirm the molecular binding of the targeted protein, Surface Plasmon Resonance (SPR) was performed with a Biacore 3000. TRAP1 recombinant protein (Abcam, ab123775) or human Hsp90 recombinant protein (HSP90AB1, Abcam, ab131923) were immobilized on a CM5 sensor chip surface using the amine coupling method according to the manufacturer’s instruction (Biacore, GE Healthcare). CVM-1125 was dissolved in sodium carbonate buffer (pH 9) and then diluted in HBS-EDTA-P20 buffer to desired concentrations (five serial dilutions from 1 × 10^−5^ to 1 × 10^−8^ M) and injected at flow 30 μL/min with KINJET 60/180 %pos, analyte position, volume, and dissociation time. Specific binding was calculated using BiaEval 3 software.

*In silico* exploration was performed using Accelrys Discovery Studio 2.1 software. A Protein Data Bank (PDB) format of CVM-1125 was generated using the same program. PDB for TRAP1 (PDB 4Z1G) and CVM-1125 were subjected to CHARM force filed before using Libdock module to look for eventual binding sites. Proteins were thereafter subjected to dynamic stimulation cascade before calculating the binding energy between CVM-1125 and the target.

### Succinate level

Succinate levels in the cells were assessed using the Abcam assay kit (ab204718). Cells were grown under hypoxic conditions (1% O_2_) at 37°C using a ProOx P110 Oxygen Controller (BioSpherix) and hypoxia chamber to establish a consistent and steady expression of HIF-1α in these cells for the experiments. At 80% confluence, cells were rinsed with phosphate buffered saline (PBS; 137 mM NaCl/2.7 mM KCl/10 mM Na_2_HPO_4_/1.8 mM KH_2_PO_4_, pH 7.4), then harvested by scrapping into a modified radio-immunoprecipitation assay (RIPA) buffer (100 mM Tris-HCl, 150 mM NaCl, 1% deoxycholate, 1% Triton X-100, 0.1% SDS, pH 7.4) plus protease inhibitor cocktail (Complete Mini; Roche) using 100 μL buffer per 1 × 10^6^ cells. After sonication using a Fisher sonic dismembrator with microprobe (Fisher Scientific; five pulses at 2 s intervals) and centrifugation at 13,000 rpm for 30 min, the protein lysates (supernatants) were recovered, and protein concentrations determined using a bicinchoninic acid (BCA) assay (Bio-Rad). The samples were then deproteinated using Abcam 10 kDa Spin Columns (ab93349; which reduced the protein concentration by about 90%) and the samples were standardized at <10 μg of protein per sample per 50 μL of assay volume (as directed by the Abcam technical response team for working with these samples). The concentration of succinate (nmol/μL or μmol/mL or nM) for each sample was calculated after subtracting a background control (which was the sample run with assay buffer in place of the succinate converter component to account for and subtract any contaminating signal generated by NADH), as follows: Succinate = (A/B) × D; where: A = Amount of succinate (succinic acid) from the Standard Curve; B = Sample volume added into the reaction well (μL); D = Sample dilution factor. Two separate experiments were performed for each cell line.

### Western blotting

The methods for detecting Nodal (in its more stable ProNodal form) and Smad2/P-Smad2 proteins were as follows: 5.0 × 10^6^ human melanoma cells (C8161) were seeded into T175 culture flasks in the presence of 10 nM CVM-1118 and samples recovered for analysis at 1, 4, 8, and 24 h. Cells at 80% confluence were rinsed with PBS, harvested by scrapping into a modified RIPA buffer plus protease inhibitor cocktail using 100 μL buffer per 1 × 10^6^ cells. After sonication and centrifugation, the protein concentrations in the supernatants were determined using a BCA assay (Thermo Fisher Scientific). After addition of lithium dodecyl sulfate sample buffer (NuPage 4X LDS sample buffer; Invitrogen) and heating at 95°C for 5 min, 40 μg (for Nodal detection) or 25 μg (for Smad2/P-Smad2 detection) of protein were loaded per lane of 4%–12% Bis/Tris polyacrylamide electrophoresis gel (NuPAGE; Invitrogen) run with a MES running buffer (Invitrogen) and after separation of the proteins, the proteins electroblotted onto Immobilon-P membranes (Millipore). The blots were then blocked at 37°C with 5% bovine serum albumin (BSA) and then washed. Nodal was detected using a mouse monoclonal anti-Nodal antibody (sc-373910; Santa Cruz; 1 μg/ml in 20 mM Tris/150 mM NaCl/0.1% [w/v] Tween 20, pH 7.4 [TBST] plus 5% BSA) followed by an anti-mouse plus horseradish peroxidase (HRP) secondary antibody (NXA931; GE Healthcare; 1 μg/ml in TBST plus 5% BSA) and enhanced chemiluminescence (ECL; GE Healthcare). Smad2 was detected using a rabbit polyclonal anti-Smad2/3 antibody (07-408; Millipore; 1 μg/ml in TBST plus 5% BSA) followed by an anti-rabbit plus HRP secondary antibody (NA934V; GE Healthcare; 1 μg/ml in TBST plus 5% BSA) and ECL. The Smad2 blot was then stripped (100 mM 2-mercaptoethanol, 2% [w/v] SDS, 62.5 mM Tris-HCl, pH 6.7 at 50°C for 30 min), washed and re-probed for P-Smad2 using a polyclonal antibody (44–244G; Invitrogen; 1 μg/ml in TBST plus 5% BSA) followed by an anti-rabbit plus HRP secondary antibody (NA934V; 1 μg/ml in TBST plus 5% BSA) and ECL (IT’s Science Corporation Ltd., IT96-K004M). The Nodal and Smad2/P-Smad2 blots where then stripped and re-probed with a monoclonal mouse anti-β-actin antibody (MAB1501; Millipore; 0.5 μg/ml in TBST plus 5% BSA) followed by an anti-mouse plus HRP secondary antibody (NXA931; 0.5 μg/ml in TBST plus 5% BSA) and ECL. To assess relative changes in protein expression, the exposed films were digitized using a ChemiDoc XRS imager (Bio-Rad Laboratories) and the relative amount of protein determined against the untreated control cells normalized to a value of 1.0 using the imager’s Quantity One software package (Bio-Rad Laboratories) and corrected for loading of protein into each lane against the β-actin protein measured in the control lane.

Changes in the expression HIF-1α protein in SK-MEL28, SK-OV3, MDA-MB-231 and COLO205 cells in response to treatment with CVM-1125 was performed as follows: 5.0 × 10^6^ cells were grown under hypoxic conditions (as previously described) for 72 h, with or without 100 nM CVM-1125 in T175 culture flasks, then sub-confluent cultures harvested and whole cell protein lysates prepared and analyzed by Western blot (as above). Thirty micrograms of protein was loaded per lane of the gel for each sample followed by detection of HIF-1α protein on the final blot using a polyclonal rabbit anti- HIF-1α antibody (#3716; Cell Signaling Technologies), secondary antibody plus HRP (NA934V), then ECL. The blots were then stripped and re-probed for β-actin protein as a control (as above).

The methods for detecting TRAP1 proteins were as follows: 4 × 10^5^ SKOV-3 cells and 8 × 10^5^ COLO-205 cells were seeded onto 6-well plates. After 18 h, the culture medium was changed to contain 50, 100, 200, and 400 nM CVM-1125, and samples were recovered for analysis at 24 or 48 h. To explore the pathway of CVM-1125 degradation of TRAP1 protein, the experimental design was as follows: 1 × 10^6^ COLO-205 cells were seeded onto 6-well plates. After 18 h, the culture medium was changed to contain 200 nM CVM-1125, 25 µM chloroquine (CQ), and 2.5 µM MG132, and samples were recovered for analysis at 24 h. Cells at 80% confluence were collected with trypsin, centrifuged, and harvested by PBS into a sample buffer (1M Tris pH 6.8, 50% Glycerol, 10% SDS, 1% bromophenol blue, and 2-mercaptoethanol). Cell lysates were heated at 95°C for 10 min and loaded onto 10% SDS-PAGE. Proteins separated on the gel were transferred to a 0.45 μM PVDF membrane (Merk Milipore, IPVH00010) at 4°C overnight and was then blocked at room temperature with 5% skim milk for 30 min. The primary antibodies were diluted with 5% skim milk in PBS for β-actin (Sigma, A5441, 1:20,000) and TRAP1 (Santa Cruz, sc-13557, 1:100) and incubated for 2 h at room temperature. The PVDF membranes were then washed and incubated with an anti-mouse-IgG secondary antibody (Sigma, A9044, diluted at 1:3,000 for TRAP1, and 1:10,000 for β-actin with 5% skim milk) followed by the incubation with ECL. To assess relative changes in protein expression, the exposed films were digitized using a UVP BioSpectrum 500 Imaging System, and the Image J system was used to measure the protein expression. In cells treated with different concentrations of CVM-1125, statistical difference in relative protein expression levels was tested using Kruskal-Wallis test in COLO205 and using one-way ANOVA followed by Dunnett’s multiple comparisons test in SKOV3 cells. In COLO205 cells treated with inhibitors in addition to CVM-1125, statistical analysis was conducted using one-way ANOVA followed by Dunnett’s multiple comparisons test.

### Quantitative polymerase chain reaction (qPCR) analysis

For the qPCR analysis of Nodal and Notch expression, total RNA was isolated from cells using the PerfectPure RNA Cell Isolation kit (5Prime) and then reverse-transcribed using 1 μg RNA, RT master mix cocktail and MMLV reverse transcriptase. qPCR was performed on a 7500 Real-Time PCR System (Life Technologies) using manufacturer recommended protocol. TaqMan gene expression human primer/probe sets (Applied Biosystems) utilized were Nodal (Hs00250630_s1) and Notch4 (Hs00270200_m1). Target gene expression was normalized to appropriate house-keeping genes (e.g., RPLP0 or GAPDH). Data was analyzed using Life Technologies Sequence Detection Software.

For the expression analysis of *STK11* and *NF2* genes, total RNA was isolated from cells using QIAGEN^®^ RNeasy Plus kit and quantified using QuantStudio 3 Real-Time PCR machine. All RT-qPCR reactions were performed in a 20 μL mixture containing 100 ng of RNA template, 1X One-Step RT-qPCR Master Mix (PrimeTime^®^), and 1X qPCR Assay reagent (premixed primers and probe) (PrimeTime^®^). Target gene expression was normalized to *ACTB* and the relative RNA fold change between knockdown cell and scramble control cell was analyzed by comparative CT (ΔΔCT) analysis. Statistical significance between groups was tested using one-way ANOVA followed by Dunnett’s multiple comparisons test.

Primers and probes used are as follows:

(1) *NF2*

Probe 5′-/56-FAM/AGCAACCCA/ZEN/AGACGTTCACCGT/3IABkFQ/-3′

Primer 1 5′-TTC​CCG​CAT​GAG​CTT​CAG-3′

Primer 2 5′-GGA​GGG​CAG​TAG​ATC​TTT​TCA​TC-3′

(2) *STK11*

Probe 5′-/56-FAM/TTGTGCCGT/ZEN/AACCTCCTCAGTAGTTG/3IABkFQ/-3′

Primer 1 5′-GCC​GTC​AAG​ATC​CTC​AAG​AAG-3′

Primer 2 5′-TCG​TTG​TAT​AAC​ACA​TCC​ACC​AG-3′

(3) *ACTB*

Probe 5′-/5SUN/TCATCCATG/ZEN/GTGAGCTGGCGG/3IABkFQ/-3′

Primer 1 5′-ACA​GAG​CCT​CGC​CTT​TG-3′

Primer 2 5′-CCT​TGC​ACA​TGC​CGG​AG-3

### Whole genome CRISPR knock-out screening and validation

This study was conducted by Horizon Discovery (Cambridge, United Kingdom). COLO205 cells were transduced with whole-genome All-In-One high complexity CRISPRko library, containing eight guides per gene, at ×300 coverage. The edited population was selected with puromycin at 0.5 μg/mL for 10 days to remove any non-transduced cells ahead of screening. The screen treatment was conducted with or without 30 nM of CVM-1125, in duplicate, and maintained at 300X coverage for approximately 12 population doublings of the vehicle control condition. At screen endpoint, cell pellets were collected and gDNA was extracted using the QIAamp DNA Blood Maxi kit (Qiagen #51194). A two-step PCR protocol was used to prepare amplicons with Herculase II fusion DNA polymerase (Agilent) using custom primers based on the Illumina system followed by Kapa HiFi HotStart DNA Polymerase (Roche #KK2502) using primers from Nextera XT Index Kit v2; the amplicons were then sequenced by Next-Generation Sequencing (NGS) on an Illumina NextSeq platform. FASTQ files were evaluated for sequencing quality using the Phred scoring system for base-calling accuracy, biological quality control analysis was performed by assessing log2 fold changes of controls including non-targeting control guides, positive/essential genes control guides, and non-essential genes control guides, and then data was deconvoluted and quantified using Horizon Discovery’s proprietary mapping scripts based on published analysis tools MAGeCK robust rank aggregation (RRA) (v0.5.9.2) [33] and DrugZ (v.1.1.0.2) [34]. Unbiased hit calling was conducted by comparing samples collected from vehicle control versus drug-treated samples. The gene hits selected from CRISPR knock-out screening were analyzed with DAVID Functional Annotation Bioinformatics Microarray Analysis (ncifcrf.gov) to identify significantly enriched Kyoto Encyclopedia of Genes and Genomes (KEGG) pathways at *p* < 0.05.

Further validation of *STK11* and *NF2* by shRNA knockdown system were performed. The lentivirus containing *STK11* or *NF2*-targeting small hairpin RNA (shRNA) was prepared by the National RNAi Core Facility, Academia Sinica (Taipei, Taiwan). The shRNAs targeting *STK11*#sh1 were 5′-GCC​AAC​GTG​AAG​AAG​GAA​ATT-3′; *STK11*#sh2 was: 5′-CAT​CTA​CAC​TCA​GGA​CTT​CAC-3′; *NF2*#sh1 was 5′- TAG​TTC​TCT​GAC​CTG​AGT​CTT-3′; *NF2*#sh2 was 5′-GCT​CTG​GAT​ATT​CTG​CAC​AAT-3′. One shRNA was used as the scramble control: 5′-CCT​AAG​GTT​AAG​TCG​CCC​TCG-3′. COLO205 and HCT-116 were transfected with shRNA lentivirus and the knockdown efficiency were evaluated by q-PCR. The viability of the knockdown cells after treatment with CVM-1125 compared to the respective scramble control was evaluated using one-way ANOVA followed by Dunnett’s multiple comparisons test.

## Results

### CVM-1118 and its active metabolite, CVM-1125, have potent anticancer activity in various cancer cell lines

**FIGURE 1:**
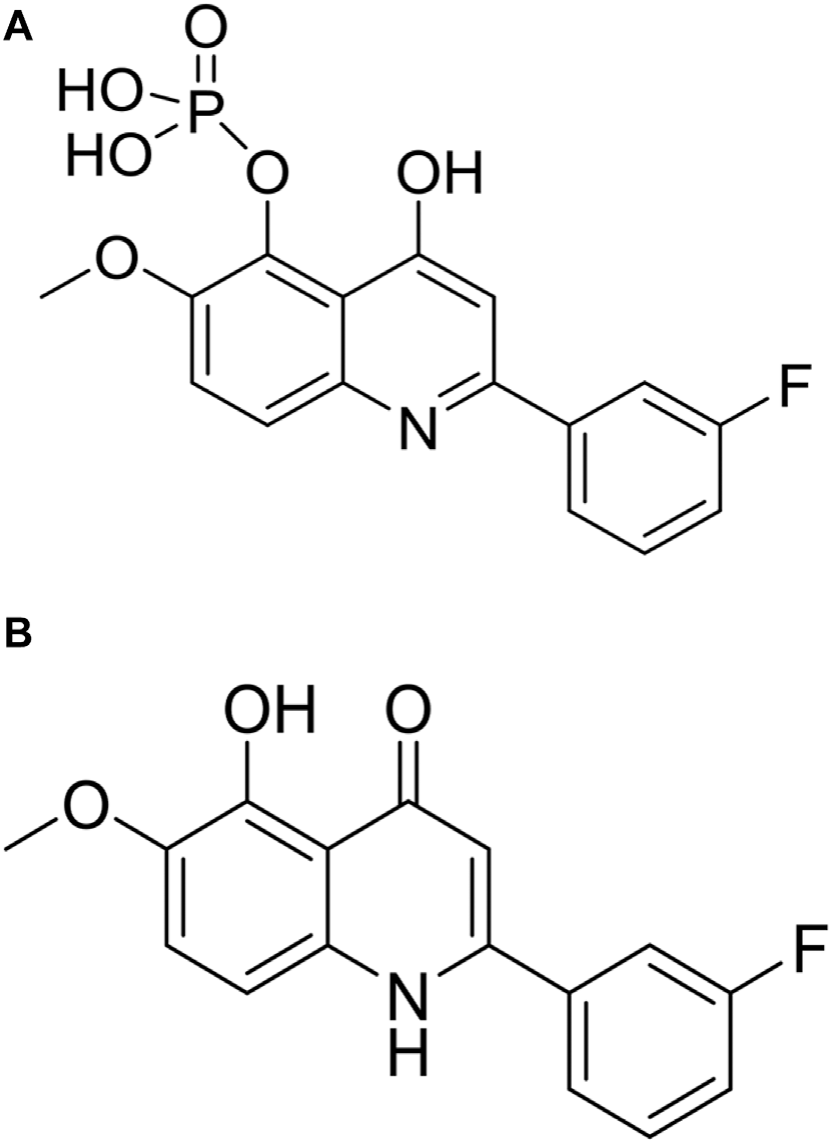
The chemical structure of CVM-1118 **(A)** with the molecular formula as C_16_H_13_FNO_6_P, and CVM-1125 **(B)** with the molecular formula as C_16_H_12_FNO_3_.

CVM-1118 (Figure 1A) is currently under development as an oral anti-cancer drug. The pharmacokinetic studies of CVM-1118 in mice, Sprague-Dawley rat and beagle dog indicated that CVM-1118 converted to CVM-1125 (Figure 1B) very rapidly following oral administration (Supplementary Figure S1; Supplementary Table S1). CVM-1125 showed growth inhibitory activity against a panel of 60 human cancer cell lines (NCI 60 screen) with over 80% of cell lines tested had GI_50_ < 100 nM. The most sensitive cell lines with GI_50_ < 50 nM were listed in Supplementary Table S2. A panel of nine selected human cancer cell lines was further used to compare the anti-proliferative activity of CVM-1118 and CVM-1125. Both compounds showed comparable potent cytotoxicity, with IC_50_ values below 50 nM, in all cell lines (Table 1). The results confirmed the potency of CVM-1118 and its active metabolite, CVM-1125, in growth inhibition and cytotoxicity for further evaluation of its potential use in cancer treatment.

**TABLE 1:** Potency of CVM-1118 and its active metabolite, CVM-1125, in human cancer cell lines.

		IC_50_ (nM)	
Cell lines	Cancer type	CVM-1118	CVM-1125
A549	Lung	26	29
DU-145	Prostate	21	27
HT-29	Colon	15	17
MDA-MB-231	Breast	18	14
MDA-MB-435^a^	Melanoma	4	5
Mia PaCa-2	Pancreatic	11	7
NCI-ADR-RES	Ovarian	14	14
SK-N-MC	Neuroblastoma	9	8
U118MG	Glioma	10	8

Cytotoxicity assays performed in nine human cancer cell lines using both CVM-1118 and CVM-1125.

^a^ MDA-MB-435 cells are now confirmed of melanoma origin and are derived from M14 melanoma cell line.

### CVM-1118 induces apoptosis and G_2_/M arrest *in vitro*

**FIGURE 2:**
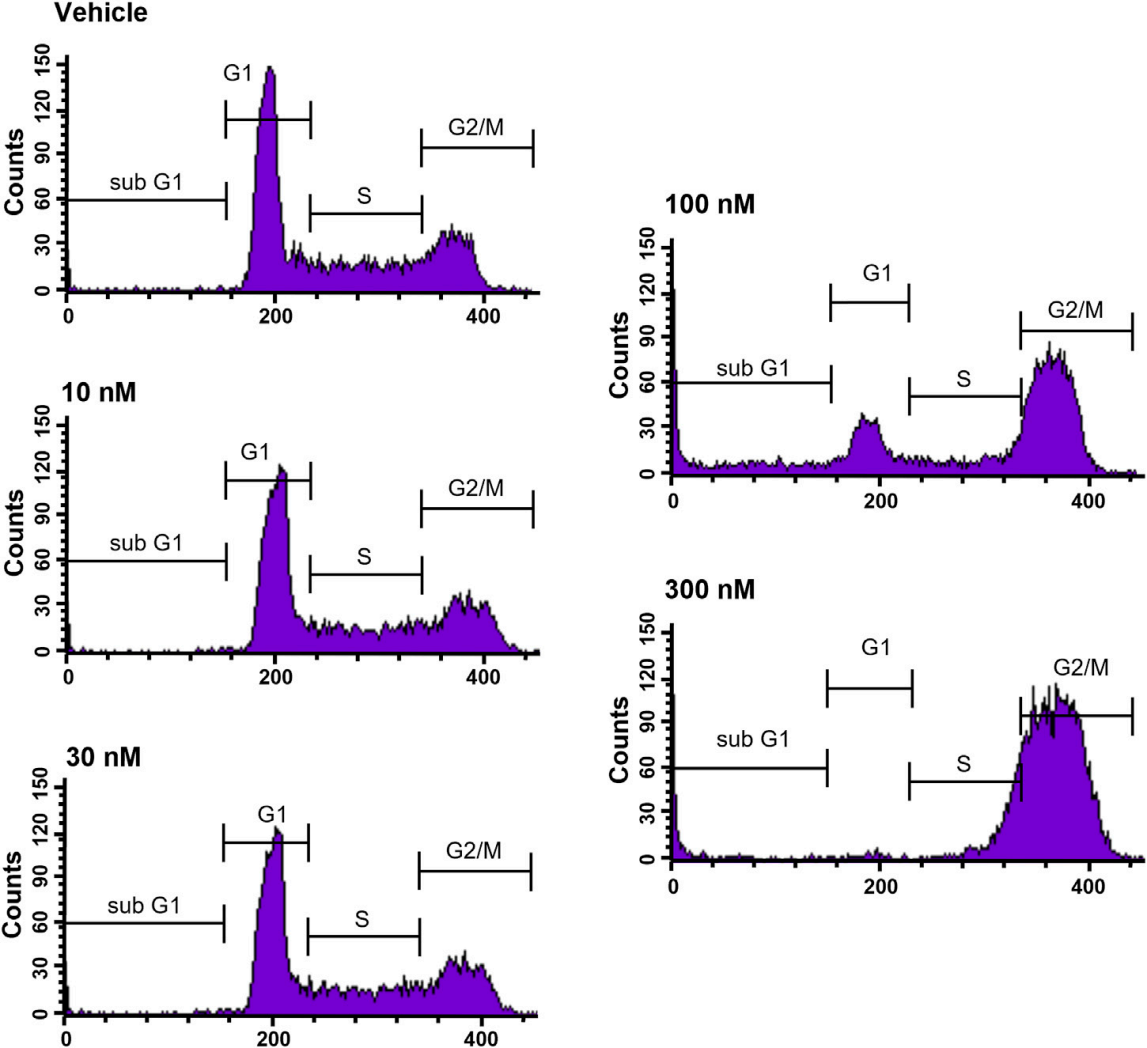
CVM-1118 induced cell cycle arrest at G_2_/M in HT29 cells. Dose-response of CVM-1118 in HT-29 cell cycle profile after 48 h treatment. Fluorescence signals of propidium iodide (FL3-A) of single cells were attributed to cell cycle phases and converted into percentage of the whole population of single cells (100%).

To further elucidate the function of CVM-1118 in cytotoxicity, cell cycle profile analysis with FACS was performed in one of the above tested cell lines, HT-29. The results showed that after 48-h treatment of CVM-1118 in HT-29 cells, there was an accumulation of cells arresting at G_2_/M phase, accompanied by a reduction of cells in G_1_ phase in a dose-dependent manner (Figure 2). The percentages of G_1_ population in 100 nM and 300 nM CVM-1118 treated cells were 16.27% and 1.58%, respectively, compared to 51.93% cells in G_1_ phase in control group (Table 2). In addition, 100 nM and 300 nM CVM-1118 treatment increased the population of HT-29 cells in sub-G_1_ phase to 26.96% and 22.46%, respectively, compared to control group (1.06%). The endoreduplicated cells (EndoR) were also increased to 2.84% after 100 nM treatment of CVM-1118 compared to the 1.36% in the vehicle control group. These results suggested a dose-dependent effect of CVM-1118 on HT-29 in the inhibition of tumor cell proliferation with a cell cycle arrest at G_2_/M phase. Apoptotic cell death indicated by the sub-G_1_ peak after treatment also occurred. Similar results in G_2_/M arrest and increased sub-G_1_ were also observed with the treatment of CVM-1125 in HCT-116 colon cancer cell line (Supplementary Figure S2).

**TABLE 2:** Effect of CVM-1118 on cell cycle distribution in HT-29 cells after CVM-1118 treatment for 48 h.

		Cell cycle profile (%)				
Test article		Sub-G_1_	G_1_ phase	S phase	G_2_/M phase	EndoR
Vehicle Control		1.06	51.93	22.17	23.91	1.36
CVM-1118	10 nM	1.24	49.34	23.02	25.66	1.28
	30 nM	1.44	50.63	23.30	23.71	1.23
	100 nM	26.96	16.27	10.90	43.42	2.84
	300 nM	22.46	1.58	23.63	50.77	2.45

### CVM-1118 inhibits human melanoma vasculogenic mimicry signaling molecules

**FIGURE 3:**
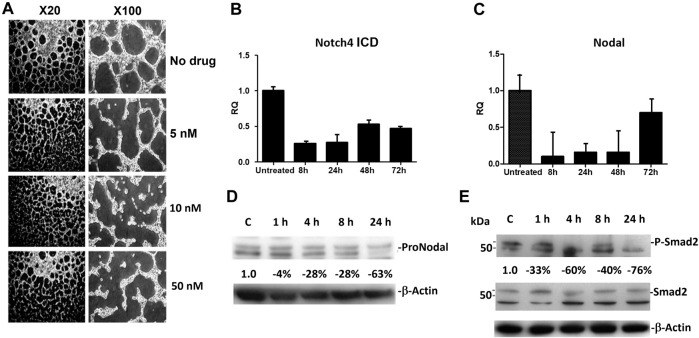
Effect of CVM-1118 on vasculogenic mimicry signaling molecules **(A)** *In vitro* standard VM assay in C8161 cells after treatment with CVM-1118 using various concentrations. ×20: 20-fold magnification. ×100: 100-fold magnification. **(B,C)** Quantitative Real Time PCR analysis of Notch4 ICD and Nodal after 10 nM of CVM-1118 treatment for 8, 24, 48, and 72 h in C8161 cells. RQ: Relative quantification of RNA expression level. **(D)** Protein level of ProNodal was determined by Western blot analysis after treatment with 10 nM CVM-1118 for 1, 4, 8, and 24 h. The percentage changes were corrected for protein loading using β-actin. **(E)** Cells were untreated (Control) or treated with 10 nM CVM-1118 for 1, 4, 8, and 24 h. The changes in the expression of Smad2 and phosphorylated Smad2 (P-Smad2) proteins (relative to β-actin) were determined by Western blot analysis. The percentage change is the reduction of protein expression ratio of P-Smad2 to Smad2 protein level relative to β-actin against the ratio in the Control cells.

Similar to the results reported previously [10], human melanoma cells VM (*in vitro*) was disrupted beginning with 5 nM of CVM-1118 treatment, and was significantly inhibited by 10 and 50 nM of CVM-1118 treatment at 24 h (Figure 3A). At the CVM-1118 concentration of 10 nM, most of the cells remained viable with the percentage of viable cells to be more than 90%. Thus, the reduction and inhibition of VM formation were the direct result of reduced capability of cells to form VM tubular networks. To understand how CVM-1118 was involved in the inhibition of VM formation mechanically, we analyzed the effect of CVM-1118 on the mRNA levels of two critical signaling proteins, Notch4 ICD and Nodal, in VM formation. The expression of Notch4 ICD and Nodal was decreased in C8161 cells treated with 10 nM of CVM-1118 for 8–72 h (Figures 3B, C). The effect of CVM-1118 on Nodal signaling was also confirmed by a 63% reduction on its protein levels after incubation with CVM-1118 for 24 h (Figure 3D). In addition, 10 nM of CVM-1118 for 24 h also resulted in 76% reduction in the ratio of phosphorylated Smad2 (P-Smad2) to Smad2 protein level (relative to β-actin) against the ratio in control (Figure 3E). Thus, CVM-1118 inhibits VM formation possibly by interfering with the Nodal signaling pathway.

### CVM-1118 inhibits formation of vasculogenic mimicry *in vivo*

**FIGURE 4:**
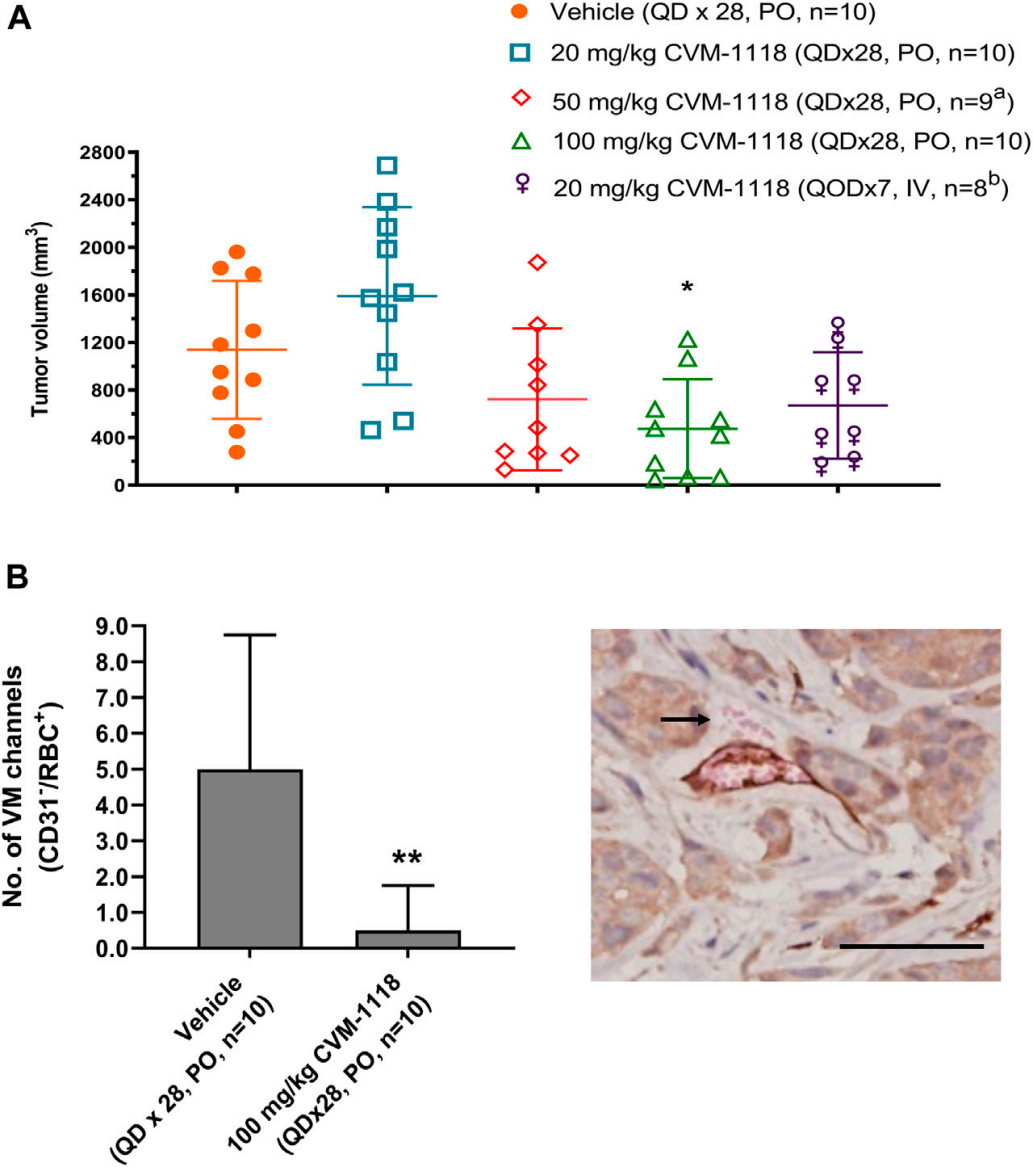
Orthotopic HCT-116 mouse xenograft model and *in vivo* vasculogenic mimicry analysis **(A)** Tumor volumes in different groups of orthotopic HCT-116 mouse xenografts on Day 43 after cell inoculation. The bars indicated the mean ± SD. **(B)** Numbers of VM (CD31^−^/RBC^+^) channels of the tumor tissues from two groups of orthotopic HCT-116 mouse xenografts treated with vehicle or 100 mg/kg of CVM-1118. Data indicate median ± interquartile range. Representative photograph shows VM presence with CD31^−^/RBC^+^ staining as indicated by arrow (×400 magnification, scale bar 20 μm). a: one animal was found dead prior to study termination. b: two animals were found dead prior to study termination. *p < 0.05, **p < 0.01 vs. vehicle.

VM inhibition by CVM-1118 was further assessed in an orthotopic mouse xenograft model using the human HCT-116 colorectal cancer cell line. HCT-116 is one of the sensitive cell lines with IC_50_ at 33 nM to CVM-1125 (Supplementary Table S2). The animals were treated with CVM-1118 orally or intravenously (i.v.). Body weight loss was observed in all the tested groups, including the vehicle control, which might be caused by the tumor development. One mouse in the 50 mg/kg treatment group, and two mice in the 20 mg/kg i.v. treatment group were found dead prior to the termination of the study which may be due to the above reason. The results showed that tumor volume of vehicle group was 1,139 ± 580 mm^3^ on day 43 post tumor inoculation. The tumor volumes were 1,591 ± 747 mm^3^, 722 ± 595 mm^3^, 475 ± 415 mm^3^, and 670 ± 447 mm^3^ for the orally 20, 50, and 100 mg/kg treatment groups, and 20 mg/kg i.v. group, respectively. CVM-1118 treatment resulted in inhibition of tumor growth, which appeared to be in a dose-dependent manner (Figure 4A). Further analysis for the numbers of VM channels revealed that CVM-1118 significantly reduced VM network formation by 85% in excised colon tumor tissues compared with that in vehicle-treated control (Figure 4B). The result further confirmed the role of CVM-1118 in reducing VM formation *in vivo*.

### TRAP1 is a direct binding target of CVM-1125—a major metabolite of CVM-1118

**FIGURE 5:**
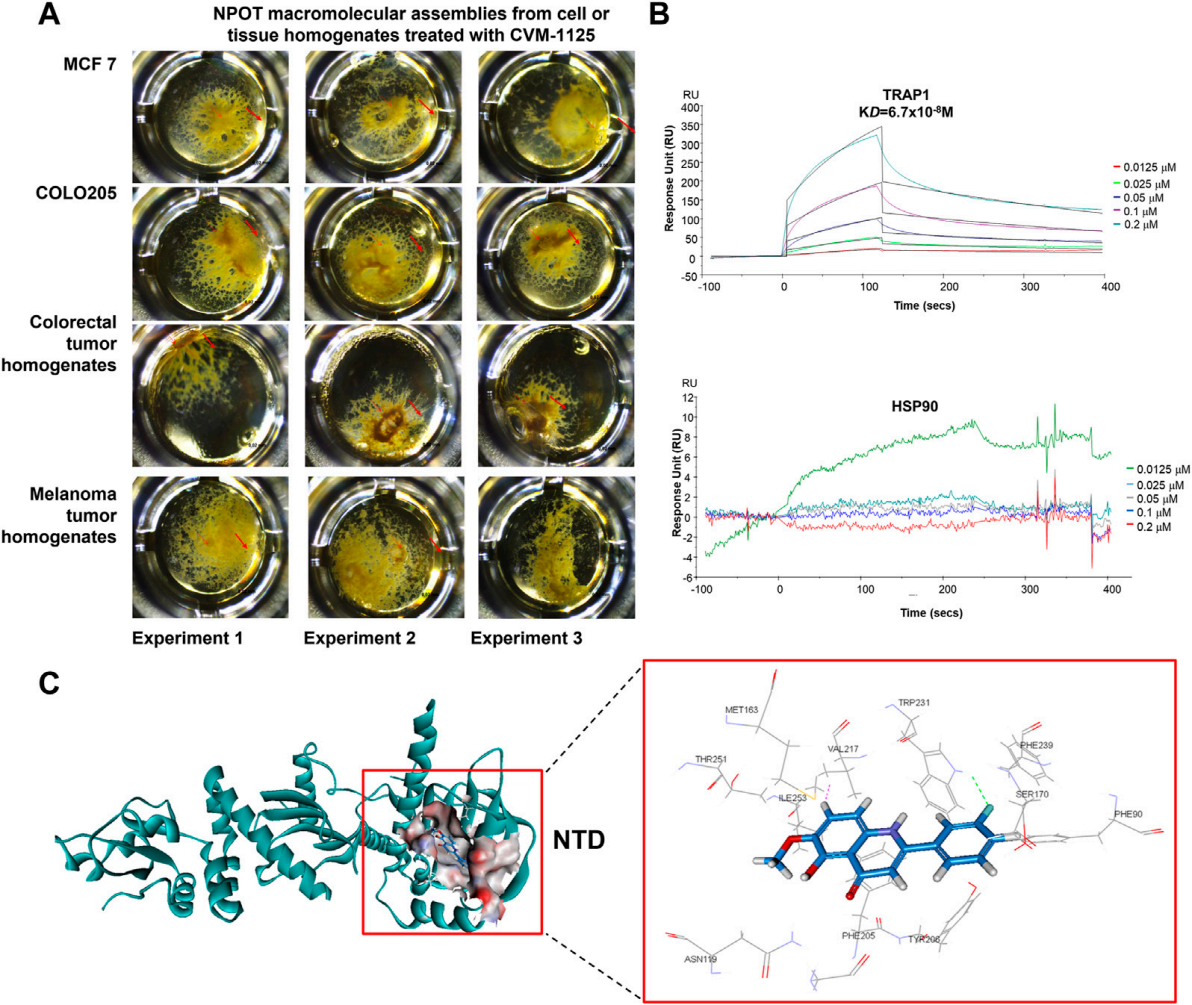
CVM-1125 binds strongly to TRAP1 **(A)** To identify CVM-1125 binding target, the NPOT assay was performed on CVM-1125 treated MCF7 and COLO205 cells, as well as the colorectal and melanoma tumor homogenates. The experiments were performed three times independently. Arrows point at the isolated drug specific macromolecular assemblies. **(B)** The binding between TRAP1 and CVM-1125 was confirmed using SPR. There is a dose-dependent interaction between CVM-1125 and the immobilized TRAP1 with *K*_*D*_ of 6.7 × 10^−8^ M. However, no specific interaction of Hsp90 to CVM-1125 was observed. **(C)** *In silico* interaction analysis of CVM-1125 with TRAP1 at the N-terminal domain (NTD).

To identify direct targets of CVM-1125, NPOT interactome analysis technology was applied to find the direct binding targets of the compound. CVM-1125 was tested in COLO205 and MCF7 cell lines and tissues of human colorectal cancer and melanoma patients (Figure 5A). Mass spectrometry analysis using human proteome database identified 52 different proteins: 30 of which were shared among the four samples (Supplementary Table S3), whereas 22 of which were specific for colorectal cancer clinical tissues (Supplementary Table S4). Eleven proteins which were functionally related to cancer were chosen to confirm the binding via SPR, including: 1) Carcinoembryonic antigen-related cell adhesion molecule 5 (CEACAM5); 2) Transforming growth factor-beta-induced protein ig-h3 (TGFβig-h3); 3) Integrin beta-4; 4) Catenin alpha-2; 5) Integrin beta-2 (CD18); 6) Transforming growth factor beta-1 induced transcript 1 protein (TGFβ1/1) (HIC5); 7) Growth arrest and DNA damage-inducible proteins-interacting protein1 (GADD45GIP1); 8) Catenin beta-1; 9) Nucleolin; 10) TNF receptor associated protein 1 (TRAP1); and 11) Renin receptor (ATP6IP2).

SPR experiments revealed that a dose-dependent interaction exists between immobilized recombinant human TRAP1 protein (Abcam, ab123775) and CVM-1125. The dissociation constant (*K*_*D*_) between TRAP1 protein and CVM-1125 was estimated to be 6.7 × 10^−8^ M (Figure 5B), which was the strongest binding affinity of the 11 selected candidates (Table 3). Moreover, *in silico* analysis demonstrated that CVM-1125 interacts with TRAP1 at the N-terminal domain. The interaction pocket is comprised of 11 amino acids all in 10 Å distance from CVM-1125. These amino acids are Phe90, Asn119, Met163, Ser170, Phe205, Tyr206, Val217, Trp231, Phe239, Thr251, and Ile253. *In silico* docking analysis showed at the flourophenyl ring of CVM-1125, it interacts with Trp231 through a hydrogen bond. The hydrogen from CVM-1125 within quinolin forms electrostatic interaction with Val217 (Figure 5C). Since CVM-1125 was shown to bind to the ATP binding pocket of TRAP1, it is speculated that CVM-1125 also binds to other proteins within the Hsp90 family. Thus, SPR analysis using recombinant human Hsp90 beta protein (Abcam, P08238) was performed; however, no specific interaction to CVM-1125 was observed (Figure 5B). This result indicates that CVM-1125 binds to TRAP1 specifically, but not universally to any other heat-shock proteins.

**TABLE 3:** Kinetic and affinity constants (*ka*, *kd*, and K_D_) to CVM-1125 determined by SPR.

No.	Protein	Association (*ka*) M^−1^s^−1^	Dissociation (*kd*) s^−1^	K_D_ (*kd/ka*) M
1	TRAP1	3.02 × 10^4^	2.2 × 10^−3^	6.68 × 10^−8^
2	ATP6IP2	1.22 × 10^5^	2.1 × 10^−2^	1.73 × 10^−7^
3	CD18	14.4	1.08 × 10^−5^	7.48 × 10^−7^
4	Nucleolin	1.35 × 10^4^	1.22 × 10^−2^	9.06 × 10^−7^
5	Catenin α2	No dose dependent interaction		
6	Integrin β4	No dose dependent interaction		
7	CEACAM5	No interaction		
8	TGFβ1/1	No dose dependent interaction		
9	GADD45GIP1	No interaction		
10	Catenin β-1	No interaction		
11	TGFβig-h3	No interaction		

### CVM-1125 reduces TRAP1 protein level *in vitro*

**FIGURE 6:**
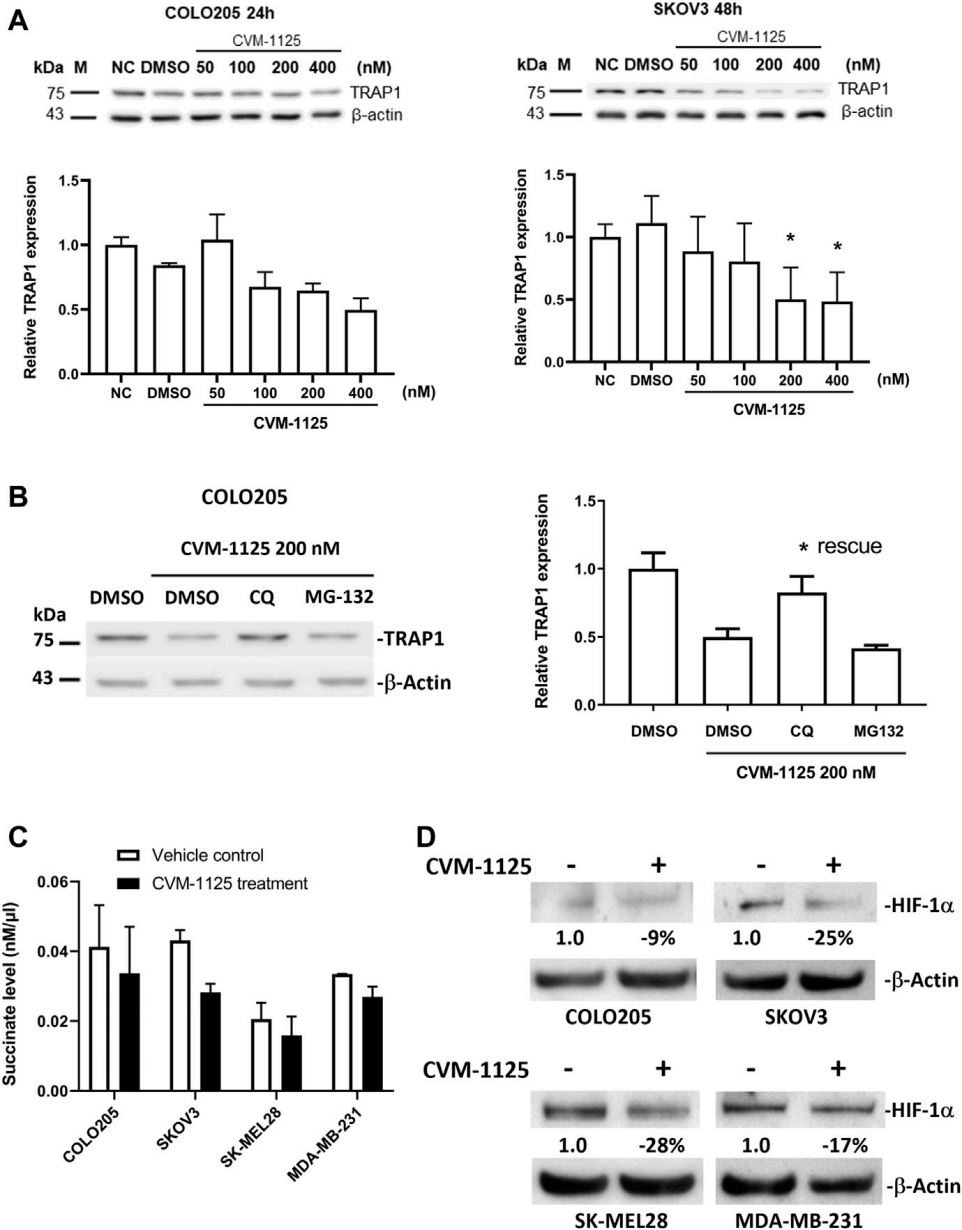
Changes of TRAP1, HIF-1α and succinate levels after CVM-1125 treatment **(A)** TRAP1 protein levels, with or without CVM-1125 treatment, in COLO205 and SKOV3 cell lines. The protein level of TRAP1 was examined by Western blot and the relative expression at different treatment concentration was calculated by normalization to β-actin and normal control (NC) set as 1.0. Data represent median ± interquartile range for not normally distributed data in COLO205 cells and mean ± SD for normally distributed data in SKOV3 cells. **(B)** TRAP1 protein levels after treatment with CVM-1125 and chloroquine (CQ) lysosome inhibitor or proteasome inhibitor (MG132) in COLO205 cell line. Relative TRAP1 protein level was calculated by normalization to β-actin and DMSO vehicle control set as 1.0 and is shown in right. Significant difference (* = *p* < 0.05) is shown in the CQ treatment group that rescues the inhibition of TRAP1 expression by CVM-1125 treatment alone (*n* = 3). **(C)** Succinate levels without (Vehicle control) or with CVM-1125 treatment in SK-MEL28, MDA-MB-231, COLO205, and SKOV3 cell lines. **(D)** HIF-1α protein levels without or with CVM-1125 treatment in SK-MEL28, MDA-MB-231, COLO205, and SKOV3 cell lines. The relative amount of HIF-1α protein level was calculated against the untreated control cells normalized to a value of 1.0 after correcting for loading of β-actin protein measured in the control lane.

To further investigate the impact of CVM-1125 on TRAP1, the protein level of TRAP1 was analyzed in COLO205, as well as in an ovarian cancer cell line SKOV3 after CVM-1125 treatment. TRAP1 protein levels were reduced with increasing concentrations of CVM-1125 in both COLO205 and SKOV3 cells (Figure 6A). To further clarify how CVM-1125 affects TRAP1 protein, COLO205 cells were co-treated with CVM-1125 and either the lysosomal inhibitor, chloroquine, or the proteasome inhibitor, MG132. The results show that treatment with CVM-1125 alone inhibited the expression of TRAP1 and this effect was abrogated to the same level of vehicle control only in cells co-treated with chloroquine but not with MG132 (Figure 6B). This data demonstrated that the decrease of TRAP1 protein level by CVM-1125 was mediated by the lysosomal degradation system. We further investigated the downstream effect of TRAP1 inhibition. The levels of succinate with or without CVM-1125 treatment in COLO205 and other cell lines were measured. The reduction of succinate levels was observed after treatment with 100 nM CVM-1125 for 72 h–by 18%, 35%, 23%, and 20% in COLO205, SKOV3, SK-MEL28, and MDA-MB-231 cells, respectively, compared to the respective vehicle control (Figure 6C). Consistently, the reduction of HIF-1α protein levels was also observed after treatment with 100 nM CVM-1125—by 9%, 25%, 28%, and 17% in COLO205, SKOV3, SK-MEL28, and MDA-MB-231 cells, respectively (Figure 6D). These results, along with the concurrent observation of a reduction in the accumulation of cellular succinate levels after treating the cells with CVM-1125, suggest that a reduction in accumulated cellular succinate levels may, in part, contribute to the destabilization of HIF-1α.

### Identification of pharmacogenomic biomarkers of CVM-1125

**FIGURE 7:**
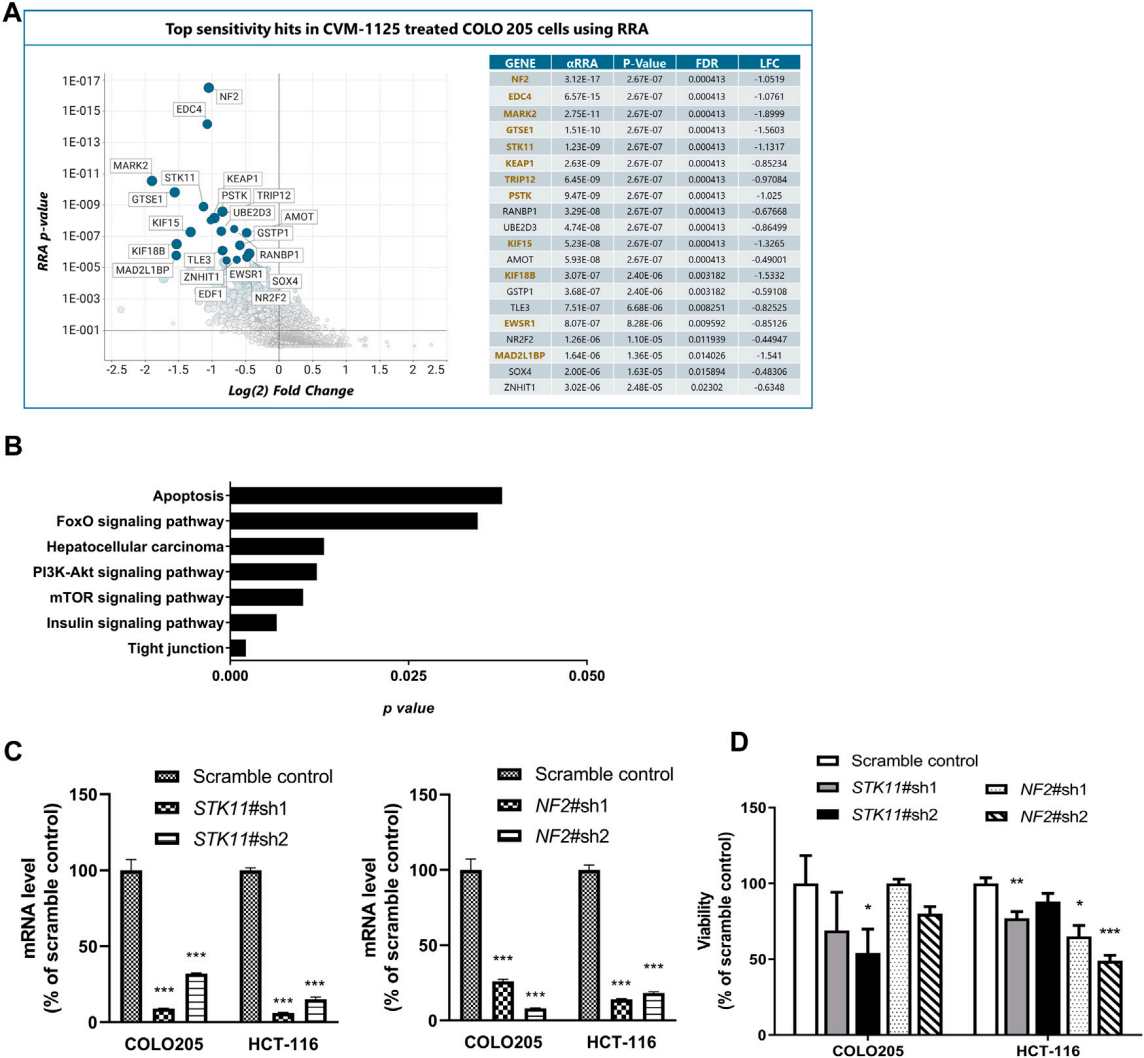
Identification of Pharmacogenomic Biomarkers of CVM-1125 **(A)** Top sensitivity hits to CVM-1125 (ranked by RRA adjusted *p*-value) are labelled by gene name in the graph on the left and individual metrics are listed in the table on the right. Hit overlap with DrugZ analysis is indicated by gene names in gold. (αRRA: a modified robust rank aggregation algorithm; FDR: false discovery rate; LFC: Log2 of fold change) **(B)** KEGG pathway enrichment was carried out with the top hits. The *p*-value (bar) for each pathway was shown. **(C)** Quantitative PCR analysis of *STK11* or *NF2* mRNA expression in scramble control or shRNA knockdown of COLO205 and HCT-116 cells after approximately 48 h transfection. **(D)** Cell viability was determined by Alamar Blue assay in COLO205 and HCT-116 cells treated with 50 nM CVM-1125 for 72 h. For each bar, the fluorescence of CVM-1125-treated cells was first normalized to the respective vehicle control, then normalized to the scramble control of COLO205 or HCT-116, respectively and presented as percentage viability as shown. Bars indicate the mean ± SD of triplicate analyses. *p < 0.05, ***p < 0.001 vs. scramble control.

To identify pharmacogenomic biomarkers of CVM-1125, a whole genome CRISPR knockout screening was performed in COLO205 cells to identify loss of function perturbations that enhance sensitivity of cancer cells to CVM-1125. The result showed that several hits significantly enhanced the cellular sensitivity to CVM-1125 (Figure 7A). There are 86 common gene hits from the top 100 sensitive and resistance genes obtained by DrugZ and RRA analysis (Supplementary Table S5). We thus further performed KEGG pathway enrichment analysis with these hits. The results indicated that tight junction, mTOR, insulin, PI3k-AKT, hepatocellular carcinoma, FoxO, and apoptosis pathways (*p* < 0.05) were the possible druggable signaling pathways of CVM-1125 (Figure 7B). From these hits, we further validated two tumor suppressor genes, *STK11* and *NF2*, using shRNA knockdown in COLO205 and HCT-116. The data show that even in these highly CVM-1125-sensitive cancer cell lines, silencing of *STK11* or *NF2* (Figure 7C) was able to induce further reduction in cell viability compared with scramble control (Figure 7D). These results indicate that *STK11* and *NF2* are likely major pharmacogenomic biomarkers of CVM-1118.

## Discussion

CVM-1118 is a novel small-molecule drug, currently under Phase 2a clinical development for cancer treatment. Our investigation focused on assessing the effects of CVM-1118 in inhibiting cancer growth and tumor cell vasculogenic mimicry. It was important to use various cell lines to determine if the mechanistic targets we observed were cell line specific or universal in nature. Since CVM-1118 has been shown to be rapidly and completely metabolized to form active CVM-1125 following administration in animals, further biological tests were conducted using CVM-1125. The cytotoxicity related studies using various cell lines was to demonstrate the ability of CVM-1118/1125 in treating different cancer types. Overall, our study results show that CVM-1118 at low concentrations exhibited not only anti-proliferation and pro-apoptosis activities but also prominent activity in the inhibition of VM formation and VM signaling molecules critical for network tubulogenesis. To further elucidate the molecular mechanism of CVM-1118 as a novel anti-cancer agent, the potential binding targets of CVM-1118 were identified by the NPOT method. Further validation with SPR showed that TRAP1, also named as Hsp75, has the strongest binding affinity to CVM-1125. The binding of CVM-1125 to TRAP1 was also confirmed in a dose-dependent manner.

The mechanism of action of CVM-1125 targeting TRAP1 was examined. A previous report indicated that TRAP1 promotes tumorigenesis by binding to and inhibiting succinate dehydrogenase (SDH), the complex II of the respiratory chain, which subsequently elicits the stabilization of the proneoplastic transcription factor HIF-1α, as a result of succinate accumulation [35]. It was found that CVM-1125 reduced TRAP1 protein level by the lysosomal degradation system and suppressed the downstream signaling of TRAP1 by inhibiting SDH activity to reduce succinate levels, which then increased prolyl hydroxylase activity leading to destabilization of HIF-1α, an important regulator for neoplastic growth and VM formation. Recent studies have demonstrated that a high level of succinate in the tumor microenvironment is an active factor promoting tumorigenesis [36] and cancer metastasis [37]. Thus, developing compounds such as CVM-1118 by targeting the binding to TRAP1 will help reduce the succinate accumulation and prevent tumor progression. It is known that overexpression of TRAP1 exerts its protective function in cancer cells by inhibiting the activation of CypD [38]. Blocking the function of TRAP1 may lead to opening of the mitochondrial permeability transition pore (mPTP) and release cytochrome c from mitochondria into the cytosol and activate the caspase kinase cascade which then leads to cell apoptosis [39]. Previous study showed that using Human Apoptosis Array Kit to measure the expression level of 35 apoptosis-related proteins in CVM-1118 treated cells, the expression levels of eight proteins were elevated with the largest changes in cleaved caspase-3, p27, p21, and phosphorylated p53 [10]. This study further provides the evidence that CVM-1125 binds to TRAP1, which may interfere the interaction of TRAP1 with CypD to negate its protective function, and subsequently induce mitochondrial apoptosis in cancer cells. Therefore, the reduction of TRAP1 protein levels by CVM-1125 may act to obstruct the downstream signaling axis, which contributes to the inhibition of tumor growth and the induction of apoptosis via caspase-3.

Additionally, our previous data showed that CVM-1118 exhibited a distinct ability to reduce VM tubular and branching structures in cancer cells [10]. Here, the *in vivo* data confirmed that CVM-1118 significantly inhibited VM formation in a mouse orthotopic xenograft model of human colon cancer. TRAP1 is known to engage in hypoxia-associated signaling pathways, particularly affecting HIF-1α, a critical transcription factor responsible for turning on the expression of genes regulating VM formation, e.g., Nodal, VEGF-A, VEGFR1, EphA2, and Twist1. Thus, hyper-expression of TRAP1 facilitates the formation of VM. HIF-1α also stabilizes the Notch intracellular domain (NICD) protein and subsequently activates genes with Notch-responsive promoters, including Nodal. Notch4 and Nodal have been identified as two critical signaling proteins in metastatic melanoma which contribute to VM formation [40]. By inhibition of the Nodal expression, the phosphorylation of Smad2 is decreased and the Smad2-mediated downstream pathway can be inhibited [41]. Here, we confirmed that treating tumor cells with CVM-1125 reduced the downstream factors of TRAP1 including HIF-1α, Nodal, and Notch. In summary, current data support the actions of CVM-1125 on reduction of VM formation via downregulating HIF-1α by targeting TRAP1.

TRAP1 has been regarded as a potential therapeutic target for cancer treatment. Several active compounds were developed as inhibitors for blocking TRAP1’s chaperon activity by targeting the ATP binding pocket, such as 17-AAG, Gamitrinib, SMTIN-P01 and DN401 [42–44]. However, proteins within the heat shock protein family share the same protein domain structure, including the ATP binding pocket at the N-terminal domain [45]. These investigational compounds share the same inhibitory activity on Hsp90 due to targeting the highly conserved domain among the HSP family members. Although the *in silico* analysis indicated that CVM-1125 bound to the ATP binding pocket of TRAP1, SPR analysis demonstrated CVM-1125 was not interacting with Hsp90, and the *in vitro* ATPase assay showed no influence on the ATPase activity of TRAP1 (data not shown). Therefore, CVM-1118 is expected to cause fewer off-target effects in clinical applications compared with other TRAP1 inhibitors.

To further explore the usage of CVM-1118 in precision medicine, we performed whole genome CRISPR knockout screen to identify potential pharmacogenomic biomarkers. Several genes were found to be associated with significant improvement of the drug sensitivity in relevant knockout cells. Further analysis of these potential biomarkers by KEGG showed two signaling pathways, mTOR and PI3K-AKT, which were also identified as CVM-1118 druggable signaling pathways in previous work [10]. Here, we validated the two tumor suppressor genes, *STK11* and *NF2*, involved in the mTOR signaling pathway which have been suggested as promising oncogenic therapeutic targets in recent studies [46, 47]. *STK11*, also named *LKB1*, controls the activity of AMP-activated protein kinase (AMPK) by phosphorylation and downregulates mTOR signaling, thereby inhibiting cancer cell proliferation and protein synthesis [48]. *STK11* is also a central regulator of tumor metabolism through the regulation of HIF-1α-dependent metabolic reprogramming [49]. In NSCLC, S*TK11* is the third commonly mutated gene (16% in the Caucasian population) and found to co-mutate with KRAS in 12% of patients [50]. Patients with *STK11*/*KRAS* co-mutation are more resistant to conventional therapies and immunotherapy. Thus, new drug treatment for these patients is needed [51]. Interestingly, in the NCI 60 assay, CVM-1125 exhibited high cytotoxicity in the two NSCLC cancer cell lines (A549 and H460) carrying *STK11* loss-of-function mutation [10, 52]. Similarly, another tumor suppressor gene, *NF2*, was validated as a potential biomarker of CVM-1118. In human schwannomas and meningiomas, loss of *NF2* leads to activation of PI3K/AKT/mTOR and thereby inhibits cancer cell proliferation [53]. It is known that TRAP1 can overcome metabolic stress and promote tumor cell metastasis by inhibiting the activation of AMPK and then activate mTOR signaling [54]. This suggests CVM-1118 may have the potential to treat cancer patients carrying *STK11*- or *NF2*-deficient mutation–via targeting TRAP1 and inhibiting the activation of mTOR/AKT signaling pathways.

**FIGURE 8:**
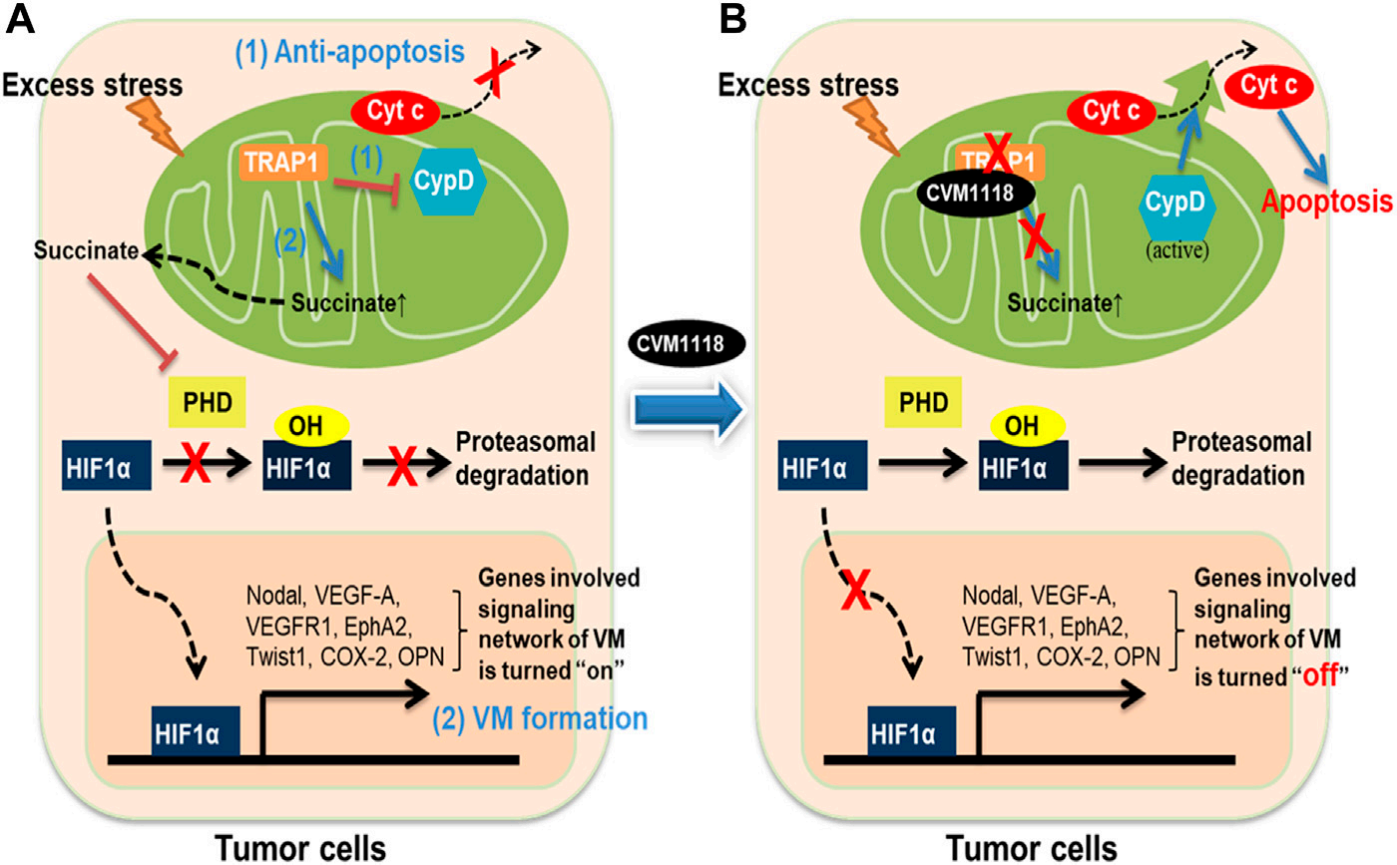
Schematic diagram showing the anti-tumor mechanisms of CVM-1118 via targeting TRAP1. Molecular mechanisms in tumor cells to show how TRAP1 mediates anti-apoptosis and promotes VM formation **(A)** and the effects of CVM-1118 treatment **(B)**.

In conclusion, these data allow us to propose a mechanism of action for CVM-1118 in the suppression of tumor growth, induction of cell apoptosis, and inhibition of VM formation–via targeting TRAP1 (Figure 8). Here, we found that the action of CVM-1125 on inhibiting TRAP1 protein level impeded succinate accumulation and led to destabilization of HIF-1α. These findings clearly support CVM-1118 as a novel TRAP1 inhibitor with distinctive activity in inhibiting VM formation and is thus considered a promising anti-cancer therapeutic. In addition, from the identified pharmacogenomic biomarkers of CVM-1118, treating cancer patients carrying specific mutations that activate signaling pathways downstream of TRAP1, such as mTOR and AKT, is expected to have a greater drug response that can be further tested in future clinical trials.

## Data Availability

The original contributions presented in the study are included in the article/Supplementary Material, further inquiries can be directed to the corresponding author.
