# Supplementary material for: CVM-1118 (foslinanib), a 2-phenyl-4-quinolone derivative, promotes apoptosis and inhibits vasculogenic mimicry via targeting TRAP1
Source: Pathol Oncol Res. 2023 Jun 7;29:1611038. doi: 10.3389/pore.2023.1611038 (PMC10283505; doi:10.3389/pore.2023.1611038)
Supplement: Supplementary file 1 [file DataSheet7.PDF]

Supplementary Table S5

Gene list of overlapping 50 sensitive hits and 36 resistant hits from top 100 hits identified by DrugZ and RRA analysis

Sensitive hits		Resistant hits	
AHNAK	MAPRE1	ACTG1	RAF1
AMOT	MARK2	ALOXE3	RBL2
ANKRD52	NECAB3	ASB7	RBM22
ARMC5	NF2	BCL2L2-PABPN1	SARAF
CD46	NR2F2	BRF1	SLFN14
CFL1	POLDIP3	BTNL8	STK10
CTSF	PRG2	CHURC1	TAZ
EDC4	PSTK	EVPLL	THEM4
EDF1	RANBP1	F13A1	TIMM23
EEFSEC	RCE1	FLOT2	USO1
EIF4E2	SAFB	FUT8	ZNF654
ENPP7	SEC24A	GALNT1	
EWSR1	SGO2	GJA4	
FAM118B	ST6GALNAC4	GOSR2	
FAM83D	STK11	IDI2	
GSG2	SUPT3H	IFNW1	
GSK3B	TLE3	IRG1	
GSTP1	TMEM50A	KIF2C	
GTSE1	TNNI2	MAPK6	
HMMR	TRIP12	MRPS31	
KEAP1	TRNT1	MZF1	
KIF15	TUBA1C	NEXN	
KIF18B	UBE2D3	NOX5	
KRT8	ZNHIT1	PERP	
MAD2L1BP	ZWILCH	PRKAA2	
