# Supplementary material for: CVM-1118 (foslinanib), a 2-phenyl-4-quinolone derivative, promotes apoptosis and inhibits vasculogenic mimicry via targeting TRAP1
Source: Pathol Oncol Res. 2023 Jun 7;29:1611038. doi: 10.3389/pore.2023.1611038 (PMC10283505; doi:10.3389/pore.2023.1611038)

Figure S2 CVM-1125 induced cell cycle arrest at G_2/M in HCT-116 cells
Dose-response of CVM-1125 on cell cycle profile in HCT-116 cells was assessed by flow cytometry after treatment for 48 hours. Fluorescent signals of propidium iodide (FL2-A) of single cells were attributed to cell cycle phases and converted into percentage of the whole population of single cells (100%). The data shown are representative of at least three independent experiments.

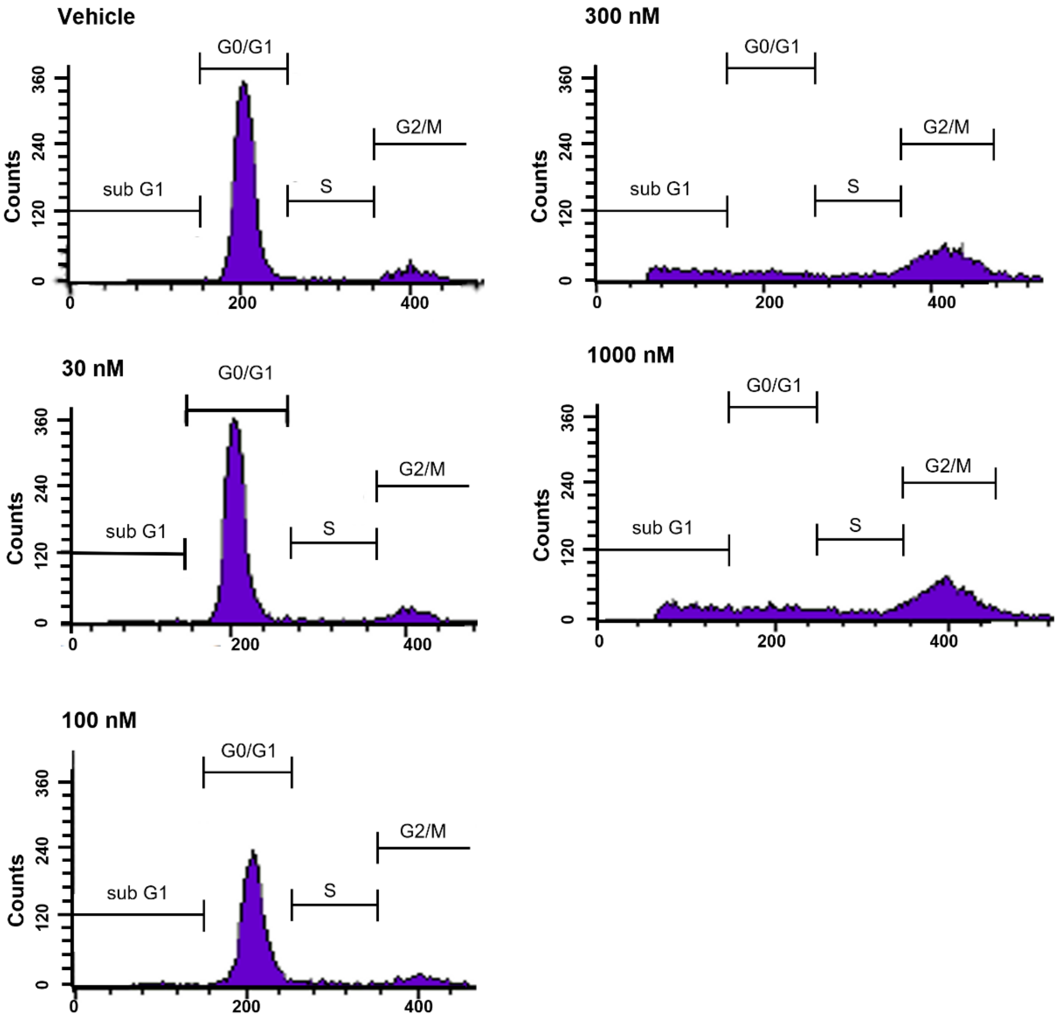

Supplement: Supplementary file 2 [file DataSheet2.PDF]
