# Supplementary material for: CVM-1118 (foslinanib), a 2-phenyl-4-quinolone derivative, promotes apoptosis and inhibits vasculogenic mimicry via targeting TRAP1
Source: Pathol Oncol Res. 2023 Jun 7;29:1611038. doi: 10.3389/pore.2023.1611038 (PMC10283505; doi:10.3389/pore.2023.1611038)
Supplement: Supplementary file 3 [file DataSheet4.PDF]

Supplementary Table S2

The most sensitive cell lines to CVM-1125 with GI_{50} < 50 nM in the NCI 60 cytotoxicity screening

Ranking	Tissue type	Cell Line	GI_{50} (nM)
1	Melanoma	MDA-MB-435	<10
2	Leukemia	SR	12
3	CNS	SNB-75	15
4	Renal	RXF 393	17.5
5	Melanoma	M14	18.9
6	Ovarian	OVCAR-3	19.1
7	Ovarian	NCI/ADR-RES	20.8
8	Prostate	DU-145	21.1
9	Leukemia	HL-60(TB)	22.2
10	Leukemia	K-562	24.1
11	Renal	A498	24.8
12	Colon	COLO 205	25.2
13	CNS	SF-295	25.5
14	CNS	SF-539	26.2
15	Ovarian	IGROV1	27
16	Breast	T-47D	28
17	CNS	SNB-19	28.8
18	Colon	HT29	29
19	Melanoma	SK-MEL-5	29.8
20	Breast	MCF7	29.9
21	Melanoma	UACC-62	31
22	Colon	HCC-2998	32.1
23	Prostate	PC-3	32.6
24	NSCLC	NCI-H460	32.8
25	Colon	HCT-116	33
26	Leukemia	RPMI-8226	33.1
27	Melanoma	UACC-257	33.7
28	NSCLC	NCI-H23	34.5
29	Leukemia	CCRF-CEM	34.8
30	Colon	HCT-15	35.5
31	Ovarian	SK-OV-3	35.6
32	NSCLC	A549/ATCC	41.4
33	CNS	U251	42.9
34	Ovarian	OVCAR-4	43.6
35	NSCLC	EKVX	46.1
36	Renal	CAKI-1	46.7
37	Leukemia	MOLT-4	47.6
38	NSCLC	HOP-62	49.6
