# Supplementary material for: CVM-1118 (foslinanib), a 2-phenyl-4-quinolone derivative, promotes apoptosis and inhibits vasculogenic mimicry via targeting TRAP1
Source: Pathol Oncol Res. 2023 Jun 7;29:1611038. doi: 10.3389/pore.2023.1611038 (PMC10283505; doi:10.3389/pore.2023.1611038)
Supplement: Supplementary file 4 [file DataSheet6.PDF]

Supplementary Table S4

List of identified proteins specific for colorectal clinical cancer tissues from mass spectrometry analysis.

Accession	Description	Sequence
sp|P14174|MIF_HUMAN	Macrophage migration inhibitory factor OS=Homo sapiens GN=MIF PE=1 SV=4	PMFIVNTNVPR-PMFIVNTNVPR
sp|P59665|DEF1_HUMAN	Neutrophil defensin 1 OS=Homo sapiens GN=DEFA1 PE=1 SV=1	YGTCIYQGR-IPACIAGER
tr|A0N5G5|A0N5G5_HUMAN	Rheumatoid factor D5 light chain (Fragment) OS=Homo sapiens GN=V<kappa>3 PE=2 SV=1	LLIYDASNR-EIVLTQSPATLSLSPGER
sp|P30453|1A34_HUMAN	HLA class I histocompatibility antigen, A-34 alpha chain OS=Homo sapiens GN=HLA-A PE=1 SV=1	WASVVVPSGQEQR-DGEDQTQDTELVETRPAGDGTFQK-AYLEGTCVEWLR-MYGCDVGPDGR-MYGCDVGPDGR-FIAVGYVDDTQFVR
sp|P01911|2B1F_HUMAN	HLA class II histocompatibility antigen, DRB1-15 beta chain OS=Homo sapiens GN=HLA-DRB1 PE=1 SV=2	SGEVYTCQVEHPSVTSPLTVEWR-HNYGVVESFTVQR-AAVDTYCR-FDSDVGEFR
sp|O75323|NIPS2_HUMAN	Protein NipSnap homolog 2 OS=Homo sapiens GN=GBAS PE=1 SV=1	SYQLRPGTMIEWGNYWAR-SGPNIYELR-KNQLLLEFSFWNEPVPR
sp|P04229|2B11_HUMAN	HLA class II histocompatibility antigen, DRB1-1 beta chain OS=Homo sapiens GN=HLA-DRB1 PE=1 SV=2	SGEVYTCQVEHPSVTSPLTVEWR-AAVDTYCR-FDSDVGEYR
sp|Q15366|PCBP2_HUMAN	Poly(rC)-binding protein 2 OS=Homo sapiens GN=PCBP2 PE=1 SV=1	QVTITGSAASISLAQYLINVR-ESTGAQVQVAGDMLPNSTER-INISEGNCPER
sp|Q9BPW8|NIPS1_HUMAN	Protein NipSnap homolog 1 OS=Homo sapiens GN=NIPSNAP1 PE=1 SV=1	MGPNIYELR-NQLLLEFSFWNEPQPR-SQMLLSR
sp|P06731|CEAM5_HUMAN	Carcinoembryonic antigen-related cell adhesion molecule 5 OS=Homo sapiens GN=CEACAM5 PE=1 SV=3	TLTLLSVTR-RSDSVILNVLYGPDAPTISPLNTSYR-SDLVNEEATGQFR-VDGNRQIIGYVIGTQQATPGPAYSGR
sp|Q04941|PLP2_HUMAN	Proteolipid protein 2 OS=Homo sapiens GN=PLP2 PE=1 SV=1	HTAAPTDPADGPV
sp|Q12805|FBLN3_HUMAN	EGF-containing fibulin-like extracellular matrix protein 1 OS=Homo sapiens GN=EFEMP1 PE=1 SV=2	SVPSDIFQIQATTIYANTINTFR-NPCQDPYILTPENR
sp|Q6XQN6|PNCB_HUMAN	Nicotinate phosphoribosyltransferase OS=Homo sapiens GN=NAPRT1 PE=1 SV=2	SGLPNFLAVALALGELGYR-AAFVAYALAFPR
sp|Q9BS92|NPS3B_HUMAN	Protein NipSnap homolog 3B OS=Homo sapiens GN=NIPSNAP3B PE=2 SV=1	VHVLWWNESADSR
sp|P16422|EPCAM_HUMAN	Epithelial cell adhesion molecule OS=Homo sapiens GN=EPCAM PE=1 SV=2	TQNDVDIADVAYYFEK
sp|Q15582|BGH3_HUMAN	Transforming growth factor-beta-induced protein ig-h3 OS=Homo sapiens GN=TGFBI PE=1 SV=1	EGVYTVFAPTNEAFR-TLFELAAESDVSTAIDLFR
sp|P09758|TACD2_HUMAN	Tumor-associated calcium signal transducer 2 OS=Homo sapiens GN=TACSTD2 PE=1 SV=3	AAGDVDIGDAAYYFER
sp|P16144|ITB4_HUMAN	Integrin beta-4 OS=Homo sapiens GN=ITGB4 PE=1 SV=5	LVFSALGPTSLR-DVVSFEQPEFSVSR-QEVEENLNEVYR-ISGNLDAPEGGFDAILQTAVCTR-VLSQLTSDYTIGFGK
sp|O43294|TGFI1_HUMAN	Transforming growth factor beta-1-induced transcript 1 protein OS=Homo sapiens GN=TGFB1I1 PE=1 SV=2	DFLQLFAPR
sp|P26232|CTNA2_HUMAN	Catenin alpha-2 OS=Homo sapiens GN=CTNNA2 PE=1 SV=5	TSVQTEDDQLIAGQSAR
sp|P05107|ITB2_HUMAN	Integrin beta-2 OS=Homo sapiens GN=ITGB2 PE=1 SV=2	TTEGCLNPR
sp|O60888|CUTA_HUMAN	Protein CutA OS=Homo sapiens GN=CUTA PE=1 SV=2	SVHPYEVAEVIALPVEQGNFPYLQWVR-TQSSLVPALTDFVR
