# Supplementary material for: CVM-1118 (foslinanib), a 2-phenyl-4-quinolone derivative, promotes apoptosis and inhibits vasculogenic mimicry via targeting TRAP1
Source: Pathol Oncol Res. 2023 Jun 7;29:1611038. doi: 10.3389/pore.2023.1611038 (PMC10283505; doi:10.3389/pore.2023.1611038)
Supplement: Supplementary file 5 [file DataSheet3.PDF]

Table S1

Pharmacokinetic parameters of CVM-1118 and CVM-1125 following a single oral administration of CVM-1118 in male CD-1 mice

**Parameter (units)**	**Parameter Value**	
**Analyte**	**CVM-1118**	**CVM-1125**
**Dose (mg/kg)**	**30**	
**Formulation**	**9% $NaHCO_3$**	
T_{max}(h)	0.25	0.25
C_{max} (ng/mL)	21	349
AUC_{0-t} (ng·h/mL)	7	279
AUC_{0-inf} (ng·h/mL)	7	287
MRT (h)	0.6	3.0
$T_{1/2}$ (h)	0.7	8.3
F (%)	2.96	-
