# Supplementary material for: CVM-1118 (foslinanib), a 2-phenyl-4-quinolone derivative, promotes apoptosis and inhibits vasculogenic mimicry via targeting TRAP1
Source: Pathol Oncol Res. 2023 Jun 7;29:1611038. doi: 10.3389/pore.2023.1611038 (PMC10283505; doi:10.3389/pore.2023.1611038)

Supplementary Results on Pharmacokinetic study of oral CVM-1118 in Mouse

Method: The pharmacokinetics of CVM-1118 and CVM-1125 were investigated in male CD-1 mice following a single oral administration of CVM-1118 at 30 mg/kg in 9% $NaHCO_3$ in fasted mice. At pre-determined time points, plasma samples were collected and stored at -20°C prior to measurement of CVM-1118 and CVM-1125 concentrations using an LC-MS/MS method. The plasma samples were collected at 0, 0.25, 0.5, 1, 2, 4, 8, 12, 24 and 27 h after dosing with 3 mice per group at each time point.

Result: For single oral administration of 30 mg/kg CVM-1118 in 9% $NaHCO_3$ solution, the C_{max} was 21 ng/mL for CVM-1118, and 349 ng/mL for CVM-1125. Bioconversion from CVM-1118 to CVM-1125 occurred very rapidly. T_{max} was 0.25 h for both CVM-1118 and CVM-1125. The drug exposure ($AUC_{0\text{-}inf}$) of CVM-1118 was low (7 ng·h/mL), indicating rapid conversion to CVM-1125 following oral dosing ($AUC_{0\text{-}inf}$ of TRX-818M1 was 287 ng·h/mL). The terminal half-life of CVM-1118 and CVM-1125 were 0.7 h and 8.3 h, respectively. The oral bioavailability of CVM-1118 was low (~3%), while the terminal half-life (8.3 h) of CVM-1125, following a single oral dose of CVM-1118, was considered favorable.

Figure S1
Mean plasma concentrations-time curves of CVM-1118 and CVM-1125 following a single dose of CVM-1118 in male CD-1 mice

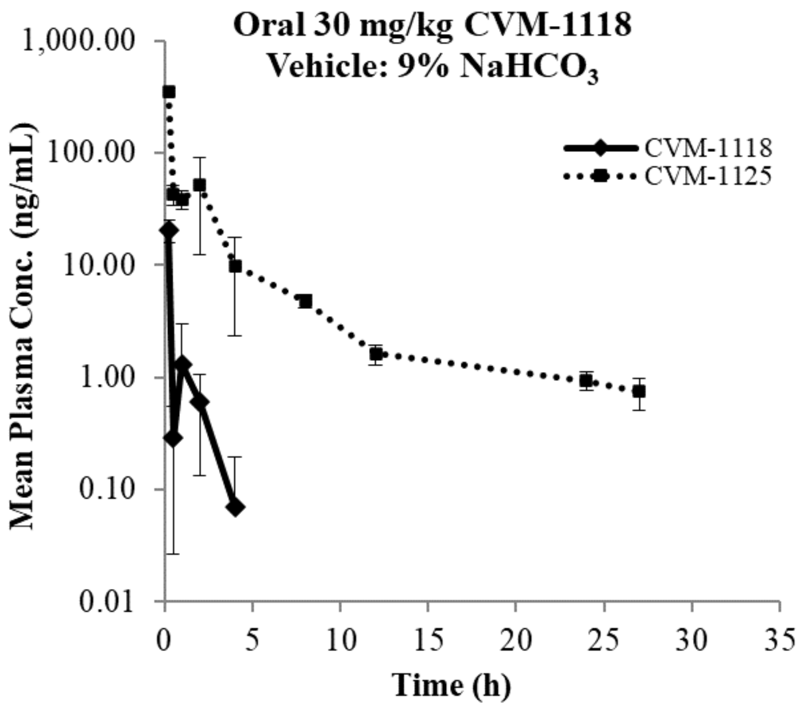

Supplement: Supplementary file 6 [file DataSheet1.PDF]
