# Supplementary material for: CVM-1118 (foslinanib), a 2-phenyl-4-quinolone derivative, promotes apoptosis and inhibits vasculogenic mimicry via targeting TRAP1
Source: Pathol Oncol Res. 2023 Jun 7;29:1611038. doi: 10.3389/pore.2023.1611038 (PMC10283505; doi:10.3389/pore.2023.1611038)
Supplement: Supplementary file 7 [file DataSheet5.PDF]

Supplementary Table S3

List of identified proteins that are common among MCF7, COLO205, colorectal and melanoma clinical cancer tissues from mass spectrometry analysis.

Accession	Description	Sequence
sp|P52597|HNRPF_HUMAN	Heterogeneous nuclear ribonucleoprotein F OS=Homo sapiens GN=HNRNPF PE=1 SV=3	VHIEIGPDGR-ATENDIYNFFSPLNPVR-MRPGAYSTGYGGYEEYSGLSDGYGFTTDLFGR-MRPGAYSTGYGGYEEYSGLSDGYGFTTDLFGR
sp|P37268|FDFT_HUMAN	Squalene synthase OS=Homo sapiens GN=FDFT1 PE=1 SV=1	EFWPQEVWSR-LFSASEFEDPLVGEDTER-QVLEDFPTISLEFR-SFAAVIQALDGEMR
sp|Q9GZT3|SLIRP_HUMAN	SRA stem-loop-interacting RNA-binding protein, mitochondrial OS=Homo sapiens GN=SLIRP PE=1 SV=1	GLGWVQFSSEEGLR
sp|Q9UBV8|PEF1_HUMAN	Peflin OS=Homo sapiens GN=PEF1 PE=1 SV=1	SGSISYTELQQALSQMGYNLSPQFTQLLVSR
sp|Q8TAE8|G45IP_HUMAN	Growth arrest and DNA damage-inducible proteins-interacting protein 1 OS=Homo sapiens GN=GADD45GIP1 PE=1 SV=1	AAALAAAVAQDPAASGAPSS
sp|Q86SX6|GLRX5_HUMAN	Glutaredoxin-related protein 5, mitochondrial OS=Homo sapiens GN=GLRX5 PE=1 SV=2	DYAAYNVLDDPELR
sp|P35222|CTNB1_HUMAN	Catenin beta-1 OS=Homo sapiens GN=CTNNB1 PE=1 SV=1	TSMGGTQQQFVEGVR
sp|Q8WY22|BRI3B_HUMAN	BRI3-binding protein OS=Homo sapiens GN=BRI3BP PE=1 SV=1	TVNTFSQSVSSLFGEDNVR
sp|P16144|ITB4_HUMAN	Integrin beta-4 OS=Homo sapiens GN=ITGB4 PE=1 SV=5	TTEGFGPEREGIITIESQDGGPFPQLGSR-LVFSALGPTSLR-MLLIENLR-DVVSFEQPEFSVSR-QEVEENLNEVYR-YEGQFCEYDNFQCPR-NDERCHLDTTGTYTQYR-ISGNLDAPEGGFDAILQTAVCTR
sp|Q7Z4H3|HDDC2_HUMAN	HD domain-containing protein 2 OS=Homo sapiens GN=HDDC2 PE=1 SV=1	STNIAAAASEPHS
sp|P06132|DCUP_HUMAN	Uroporphyrinogen decarboxylase OS=Homo sapiens GN=UROD PE=1 SV=2	LRDPEVVASELGYVFQAITLTR
sp|Q53EL6|PDCD4_HUMAN	Programmed cell death protein 4 OS=Homo sapiens GN=PDCD4 PE=1 SV=2	DSGRGDSVSDSGSDALR
sp|Q8NFH5|NUP53_HUMAN	Nucleoporin NUP53 OS=Homo sapiens GN=NUP35 PE=1 SV=1	SIYDDISSPGLGSTPLTSR
sp|Q96AE4|FUBP1_HUMAN	Far upstream element-binding protein 1 OS=Homo sapiens GN=FUBP1 PE=1 SV=3	GTPQQIDYAR-FAVGIVIGR-IGGN
sp|Q969G3|SMCE1_HUMAN	SWI/SNF-related matrix-associated actin-dependent regulator of chromatin subfamily E member 1 OS=Homo sapiens GN=SMARCE1 PE=1 SV=2	LISEILSESVVPDVR
sp|Q9UHR4|BI2L1_HUMAN	Brain-specific angiogenesis inhibitor 1-associated protein 2-like protein 1 OS=Homo sapiens GN=BAIAP2L1 PE=1 SV=2	VNNSTGTSEDPSLQR
sp|P19224|UD16_HUMAN	UDP-glucuronosyltransferase 1-6 OS=Homo sapiens GN=UGT1A6 PE=1 SV=2	SFLTAPQTEYR
sp|P26232|CTNA2_HUMAN	Catenin alpha-2 OS=Homo sapiens GN=CTNNA2 PE=1 SV=5	TSVQTEDDQLIAGQSAR
sp|Q8WUM4|PDC6I_HUMAN	Programmed cell death 6-interacting protein OS=Homo sapiens GN=PDCD6IP PE=1 SV=1	LLDEEEATDNDLR
sp|P19338|NUCL_HUMAN	Nucleolin OS=Homo sapiens GN=NCL PE=1 SV=3	LELQGPR
sp|Q6RW13|ATRAP_HUMAN	Type-1 angiotensin II receptor-associated protein OS=Homo sapiens GN=AGTRAP PE=1 SV=1	SAYQTIDSAEAPADPFAVPEGR
tr|K0A7K7|K0A7K7_HUMAN	TNF receptor-associated protein 1 (Fragment) OS=Homo sapiens GN=TRAP1 PE=2 SV=1	YESSALPSGQLTSLSEYASR-GVVDSEEIPLNLSR-ELGSSVALYSR-SAAPGSLGYQWLSDGSGVFEIAEASGVR
sp|O75787|RENR_HUMAN	Renin receptor OS=Homo sapiens GN=ATP6AP2 PE=1 SV=2	LFQENSVLSSLPLNSLSR
sp|Q96B49|TOM6_HUMAN	Mitochondrial import receptor subunit TOM6 homolog OS=Homo sapiens GN=TOMM6 PE=1 SV=1	NLSDIDLMAPQPGV
sp|P01833|PIGR_HUMAN	Polymeric immunoglobulin receptor OS=Homo sapiens GN=PIGR PE=1 SV=4	ASVDSGSSEEQGGSSR-LVSLTLNLVTR
sp|O96008|TOM40_HUMAN	Mitochondrial import receptor subunit TOM40 homolog OS=Homo sapiens GN=TOMM40 PE=1 SV=1	FVNWQVDGEYR
sp|O60361|NDK8_HUMAN	Putative nucleoside diphosphate kinase OS=Homo sapiens GN=NME2P1 PE=5 SV=1	GDFCIQVGR
sp|P54819|KAD2_HUMAN	Adenylate kinase 2, mitochondrial OS=Homo sapiens GN=AK2 PE=1 SV=2	LDSVIEFSIPDSLLIR-LAENFCVCHLATGDMLR
sp|P12277|KCRB_HUMAN	Creatine kinase B-type OS=Homo sapiens GN=CKB PE=1 SV=1	LGFSEVELVQMVVDGVK-GTGGVDTAAVGGVFDVSNADR
sp|P48426|PI42A_HUMAN	Phosphatidylinositol 5-phosphate 4-kinase type-2 alpha OS=Homo sapiens GN=PIP4K2A PE=1 SV=2	FGIDDQDFQNSLTR
